# Identification of glycosylated flavonoids as new physiological ligands of the hazel allergen Cor a 1: Complex structures reveal different binding orientation and specificity of mono- and disaccharide derivatives

**DOI:** 10.1016/j.fochx.2025.102711

**Published:** 2025-07-01

**Authors:** Julian M. Hendrich, Hatice Kara, Kristian Schweimer, Thessa P. Jacob, Maike Schneider, Susanne Baldermann, Birgitta M. Wöhrl

**Affiliations:** a Biochemistry IV – Biophysical Chemistry, University of Bayreuth, Universitätsstr. 30, D-95447 Bayreuth, Germany; b Faculty of Life Science: Food, Nutrition and Health, Food Metabolome, University of Bayreuth, Fritz-Hornschuch-Str. 9, D-95326 Kulmbach, Germany

**Keywords:** Cor a 1 isoallergens, PR-10 proteins, Glycosylated flavonoids, ITC, UHPLC/QTOF-MS/MS, NOESY, HSQC, Docking

## Abstract

The global production of hazelnuts is rising, but hazelnut allergies remain common and severe. The PR-10-like allergen Cor a 1, found in hazel pollen and nuts, consists of different isoallergens with over 67 % sequence identity. Cor a 1 binds plant secondary metabolites like the diglycosylated flavonoid Q3O-(Glc)-Gal. Understanding the ligand binding of Cor a 1 isoallergens is crucial to understand their physiological and allergenic properties.

We identified physiological mono- and diglycosylated flavonoids from hazel pollen by UHPLC/QTOF-MS and NMR spectroscopy and investigated their binding to Cor a 1 isoallergens by NMR HSQC experiments and ITC titrations. Screening analyses revealed different binding properties of isoallergens. Whereas the isoallergen Cor a 1.0501 bound only monoglycosylated flavonoids, Cor a 1.0401 also bound diglycosylated flavonoids. Protein/ligand complex structures based on NMR NOESY and computational docking experiments revealed different orientations of mono- vs. diglycosylated flavonoids in the amphiphilic pocket and specific binding of disaccharide derivatives.

## Highlights

• Cor a 1 isoallergens show different binding specificities to flavonoids.
• Glycosylated flavonoids were identified as new ligands by means of MS, NMR and ITC.
• Disaccharide flavonoid derivatives bind specifically to certain isoallergens.
• Monosaccharide flavonoids are bound in a different orientation to disaccharides.

## Graphical abstract

**Figure:**
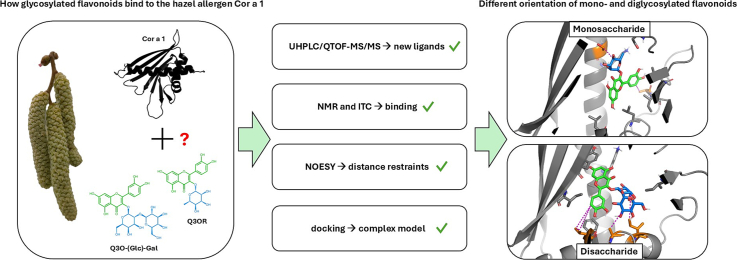

## 1 Introduction

Class 10 pathogenesis-related (PR-10) proteins are central to the plant's immune defence protecting plants against biotic and abiotic stress as well as pathogens (Jain & Kumar, 2015; Walter et al., 1990). The subfamily of PR-10-like proteins includes different allergy causing proteins from pollen and plants. The best studied member is the major birch pollen allergen Bet v 1 (Breiteneder & Kraft, 2023). PR-10-like proteins are known to trigger rhinitis and burning and itching of the tongue and oral mucosa. In general, these symptoms are not life threatening and are referred to as pollen-food syndrome (PFS) or oral allergy syndrome (OAS). However allergy induced asthma has also been described (Biedermann et al., 2019; Breiteneder & Clare Mills, 2005; Breiteneder & Ebner, 2000; Calamelli et al., 2021).

Most patients allergic to tree pollen are primarily sensitized to Bet v 1 (Lin et al., 2002). However, the IgE antibodies originally directed against Bet v 1 are known to cross-react with other PR-10 like proteins in fruits, e.g. apple, cherry, pear, vegetables and roots such as carrot or celeriac and nuts like hazel nut or stone fruits like almonds (Biedermann et al., 2019; Popescu, 2015). Cross-reaction of IgE antibodies can occur because these proteins are homologous to Bet v 1 and have a sequence identity of at least 50 % and a high structural similarity (Brazhnikov et al., 2023; Fernandes et al., 2013).

PR-10 like proteins possess a conserved three-dimensional structure consisting of a seven-stranded antiparallel β-sheet, a long C-terminal α-helix and two short helices arranged in V-shape. Together, they form a hydrophobic or amphiphilic cavity, which harbours hydrophobic as well as hydrophilic residues that can bind small molecules (Chruszcz et al., 2021; Fernandes et al., 2013; Gajhede et al., 1996; Jacob et al., 2019; Marković-Housley et al., 2003; Seutter von Loetzen et al., 2012; Seutter von Loetzen et al., 2014). PR-10 allergens were shown to bind to different chemical compounds, e.g. flavonoids, steroids or cytokinins, indicating a role in UV-protection, germination or transport of small molecules (Chruszcz et al., 2021; Jacob et al., 2019; Kofler et al., 2012; Mogensen et al., 2002; Seutter von Loetzen et al., 2014).

An important member of the PR-10 allergens is the major hazel allergen Cor a 1 from *Corylus avellana*, which also contributes to PFS since IgE antibodies against Bet v 1 can cross-react with Cor a 1. Natural PR-10 allergens like Bet v 1 and Cor a 1 exist as a mixture of different isoallergens (> 67 % amino acid sequence identity) and variants (> 90 % amino acid sequence identity) (Pomés et al., 2018). So far eight Cor a 1 isoallergens, termed Cor a 1.01 to Cor a 1.08 as well as three Bet v 1 isoallergens have been identified (Breiteneder et al., 1993; Lüttkopf et al., 2002; Hendrich et al., 2024;) (WHO/IUIS Allergen Nomenclature Sub-Committee, https://allergen.org). Understanding the structural characteristics of PR-10 allergens like Cor a 1 and their interactions with various ligands is critical for explaining their biological roles in plants and their allergenic properties in humans. While the major allergenic IgE epitopes on Cor a 1 are conformational, ligand binding could change the structural stability of the proteins or could induce conformational changes that modulate IgE recognition.

LC-MS profiles of methanolic extracts of the male flowers of *C. avellana* revealed a variety of different compounds, including diarylheptanoids as well as glycosylated flavonoids (Cerulli et al., 2018; Masullo et al., 2016). Research has shown that phenolic compounds like flavonoids are abundant in different hazel tissues, including kernels and flowers. Many of them are thought to have biological activities, such as antioxidants, UV protecting or antimicrobial agents, suggesting an important role in human nutrition and health. They are also involved in the plant's defence reactions that depend on the different sugar residues. However, to date none of these compounds have been identified as ligands of Cor a 1 proteins (Bottone et al., 2019).

Although different small molecules are bound by PR-10 allergens in vitro, so far only two natural ligands have been identified: the glycosylated flavonoid derivative quercetin-3-O-sophoroside (Q3OS) was identified as a natural ligand of the isoallergen Bet v 1.0101, and quercetin-3-O-(2′´-O-β-d-glucopyranosyl)-β-D-galactopyranoside (Q3O-(Glc)-Gal) was shown to be a natural ligand of the Cor a 1 isoallergen Cor a 1.0401 (Jacob et al., 2019; Seutter von Loetzen et al., 2014). Interestingly, the isoallergens Bet v 1.0101 and Cor a 1.0401 each selectively bind their own ligand, even though the two ligands differ only in the orientation of one OH-group in the sugar moiety (i.e. glucose vs. galactose) (Jacob et al., 2019; Seutter von Loetzen et al., 2014). Furthermore, several Bet v 1.01 variants (> 90 % amino acid sequence identity) do not bind Q3OS (Seutter von Loetzen et al., 2015). Since non-glycosylated flavonoids bind rather promiscuously to PR-10 allergens, it has been suggested that the sugar moieties are responsible for the specificity of binding (Chruszcz et al., 2021).

In a recent study we identified new Cor a 1 isoallergens and determined their localization in different plant tissues (Hendrich et al., 2024). In this work, we identified new glycosylated flavonoids extracted from natural Cor a 1 and from hazel pollen by high resolution mass spectrometry and nuclear magnetic resonance (NMR) experiments and analysed the binding properties of these compounds to the known Cor a 1 isoallergens. ^13^C/^15^N labelled Cor a 1.0401 and Cor a 1.0501 were used to identify relevant amino acid residues of the proteins for ligand binding by NMR filtered/editing nuclear Overhauser enhancement spectroscopy (NOESY) experiments.

## 2 Materials and methods

### 2.1 Cor a 1 purification

Expression and purification of recombinant (r)Cor a 1 proteins from *Escherichia coli* (*E. coli*) and purification of natural (n)Cor a 1 from hazel pollen extract was performed as previously described (Hendrich et al., 2024). The genes for Cor a 1.0401-G138S and Bet v 1-S137A were received from Eurofins Genomics (Ebersberg, Germany) and cloned into the expression vector pET-GB1a (Gunter Stier, EMBL, Heidelberg, Germany) via Gibson cloning, resulting in an N-terminal 6His-GB1a fusion of the proteins which can be cleaved off by tobacco etch virus (TEV) protease. Expression and purification was done as described for the other Cor a 1 proteins (Hendrich et al., 2024).

To obtain the protein Cor a 1.0401-3CS harbouring three amino acid exchanges: Cys5 > Ser, Cys83 > Ser and Cys161 > Ser, the corresponding wild type gene of plasmid pET11a-Cor a 1.0401 was subjected to site directed mutagenesis. The protein was purified as described (Fig. S1) (Jacob et al., 2019).

To obtain recombinant ^15^N or ^15^N/^13^C labelled proteins, *E. coli* strains were grown in M9 minimal medium supplemented with (^15^NH_4_)_2_SO_4_ (1.5 g/L), or (^15^NH_4_)_2_SO_4_ and ^13^C-glucose (2 g/L) (Jacob et al., 2019; Seutter von Loetzen et al., 2014) and purified accordingly. A purity of >95 % for the recombinant labelled and unlabelled proteins and of around 80 % for nCor a 1 was achieved (Fig. S1).

Purification of nCor a 1 was performed as previously described for nBet v 1 and nCor a 1 (Hendrich et al., 2024) For further analysis the fraction obtained by size exclusion chromatography (SEC) containing the nCor a 1/ligand complex was desalted using a PD-10 desalting column (Merck, Germany) and lyophilised. The sample was resolved in methanol, centrifuged and analysed by ultra-high performance liquid chromatography-quadrupole time-of-flight mass spectrometry (UHPLC/QTOF-MS).

### 2.2 UHPLC/QTOF-MS

The flavonoid glycosides were extracted as previously described (Harbart et al., 2023) with the following modification that the samples were redissolved in 50 μL methanol/water (1:9, *v*/v).

The hazel pollen extracts were separated by an Agilent 1260 Infinity II HPLC equipped with an Ascentis® Express F5 column (150 mm × 4.6 mm, 5 mm, Supelco, Sigma Aldrich Chemical Co., St Louis, MO, USA). Compounds were eluted using solvent A: 0.5 % acetic acid and solvent B: acetonitrile in gradient mode. The flavonoid glycosides were detected using a photodiode array detector at wavelength 370 nm as well as an Agilent 5646 LC/Q-ToF (Agilent Technologies, Waldbronn, Germany). The QToF system was equipped with a DUAL AJS ESI source (Agilent Technologies, Waldbronn, Germany) and the ions were detected in negative polarity (*m/z* 70–3200), vaporized at 350 °C at a drying gas flow of 8 L min^−1^ and nebulizer at 35 psi. The Vcap voltage was set to 3500 V and the fragmentor 175.

Additionally, to confirm the presence of quercetin 3-O-β-D-galactopyranoside (Q3OGal) in the hazel pollen extract, quercetin 3-O-β-D-glucopyranoside (Q3OGlc), Q3OGal, and the pollen extract were analysed using a Vanquish HPLC coupled to an Orbitrap Exploris 120 instrument (Thermo Fisher Scientific GmbH, Bremen) equipped with a H-EIS ion source. The chromatographic conditions were unchanged, and the source parameters were as follows: neg ionisation mode (*m/z* 100–1000, Res. 60,000), 2550 V, sheath gas 60 Arb, Aux gas 15 Arb, sweep gas 2 Arb and the ion transfer tube as well as the vaporizer temperatures were set to 350 °C.

### 2.3 Preparative purification of Q3O-(Glc)-Gal, K3OS and K3O-(Glc)-Gal by reversed phase HPLC

To obtain the potential ligands hazel pollen was extracted similarly to the procedure previously described for the extraction of flavonoids (Jacob et al., 2019). In brief, pollen was dissolved in 19 % acetonitrile, 1 % acetic acid and mixed by vortex mixer. After centrifugation at 17900 *g* for 10 min, 100 μL of the supernatant was isocratically purified by reversed phase (RP)-HPLC using an SP260/21 Nucleosil 100–7 C18 column (87 mL) (Macherey-Nagel, Düren, Germany) at a flow rate of 10 mL/min. The elution profile was analysed using the absorbance values at 255, 283 and 350 nm. Fractions containing Q3O-(Glc)-Gal or kaempferol-3-O-β-D-sophoroside (K3OS) and kaempferol-3-O-(2”-O-β-D-glucopyranosyl)-β-D-galactopyranoside (K3O-(Glc)-Gal) were lyophilized and resolved in 100 % d_6_DMSO. 1D ^1^H NMR spectra and ^13^C- heteronuclear single quantum coherence (HSQC) spectra were recorded on a 700 MHz NMR BRUKER Avance spectrometer at 25 °C to identify the molecules.

For ligand identification in the hazel pollen extract by UHPLC/QTOF-MS, for isothermal titration calorimetry (ITC), and for NMR experiments commercially purchased quercetin-3-O-α-L-rhamnoside (Q3OR), kaempferol-3-O-α-L-rhamnoside (K3OR), Q3OGlc, K3OS (BIOZOL Diagnostica in Eching, Germany) and Q3OGal (Avantor VWR) was used.

### 2.4 NMR spectroscopy

All NMR spectra were recorded at on Bruker Avance II+ 600 MHz, Avance IIIHD 700 MHz, Avance IIIHD 900 MHz, and Avance IIIHD 1000 MHz spectrometers, the latter three equipped with cryogenically cooled triple-resonance probes.

Small molecules were analysed by one-dimensional ^1^H and two-dimensional ^1^H, ^13^C HSQCexperiments in ^2^H_2_O or d_6_-DMSO. Proteins for NMR spectroscopy were dissolved in 10 mM Na-phosphate pH 7.5, 50 mM NaCl and 10 % ^2^H_2_O (NMR buffer).

For ligand screening a ligand excess of at least fivefold was chosen, as binding affinities in the range of approximately 100–200 μM were expected. According to the law of mass action, the following eq. AB = A_0_ x B/ (K_D_ + B), with A_0_ = total protein = 60 μM; B = ligand = 300 μM; was used and a K_D_ = 100 μM for the ligand was assumed. Solving the equation for AB, at a dissociation constant (K_D_) of 100 μM, 45 µM or 75 % of the protein is in complex with the ligand, meaning that chemical shift changes should be visible.

For protein resonance assignment standard double and triple resonance through-bond experiments were recorded (Sattler et al., 1999). Chemical shift perturbations were detected by ^1^H, ^15^N HSQC experiments during stepwise titration of potential ligands (30 mM stock solution in d_6_-DMSO) to uniformly ^15^N labelled protein samples in NMR buffer. Reference titrations by adding d_6_-DMSO (without ligand) to protein samples were used to observe chemical shift perturbations caused by DMSO only. Chemical shift changes of amino acid residues being in the fast exchange upon ligand titration were used to determine K_D-values_ by fitting the two-state binding model to the changes of chemical shifts during the titration using in house written MATLAB routines.

Intermolecular NOEs were observed with ^15^N/^13^C-labelled protein (300 μM) samples in ^2^H_2_O based NMR buffer with 10–15 fold excess of potential ligand using three-dimensional ^13^C-edited/^13^C-filtered NOE experiments (Zwahlen et al., 1997). To identify intraprotein NOE cross signals resulting from incomplete ^13^C labelling or incomplete ^13^C filtering the experiment was also recorded using a protein sample without ligand. NMR data were processed using TopSpin (Bruker) or in-house routines and visualised with TopSpin or NMRViewJ (Johnson & Blevins, 1994) (OneMoon Scientific, Inc.).

### 2.5 Isothermal titration calorimetry

ITC experiments were carried out on a NanoITC (TA Instruments, USA). Proteins were dialysed against 10 mM Na_2_HPO_4_/NaH_2_PO_4_, 50 mM NaCl, pH 7.5. Ligands were dissolved in the same buffer at the following final concentrations of the stock solutions: Q3OR, 3.3 mM; K3OR, 1.5 mM; Q3OGlc, 1.3 mM, and Q3OGal, 1.3 mM. All samples were centrifuged at 17900 *g* for 10 min and degassed. The initial protein concentration in the calorimeter cell was 320–340 μM in a volume of 400 μL. The thermogram was recorded at 25 °C using 2.0 μL injection volume of the ligand solution, an injection interval of 200 s and a stirring rate of 300 rpm. Buffer titrations were used as a blank and subtracted from the measurements. To determine K_D_-values, curves to the data sets were fitted with an independent binding model using the program NanoAnalyze Data Analysis Version 4.0.0.4 (TA Instruments, USA).

### 2.6 Bioinformatic tools

Pairwise sequence alignments were performed with EMBOSS needle and multiple sequence alignments with Clustal Omega (Madeira et al., 2019) and by SnapGene software (from Insightful Science; available at snapgene.com).

### 2.7 Complex structure model

The HADDOCK 2.4 web server was used to dock Q3OR and Q3OGlc to Cor a 1.0501 and Q3O-(Glc)-Gal to Cor a 1.0401 (Honorato et al., 2021, Honorato et al., 2024). The structure and PDB file of Cor a 1.0501 were generated by AlphaFold2 (Jumper et al., 2021). For Cor a 1.0401 the PDB file 6GQ9 was used. The SMILES codes of the ligands were generated by ChemDoodle 6.0 (Todsen, 2014) and their PDB files were generated with NovoPro (NovoPro Bioscience Inc). The structure of Q3OR, Q3OGlc and Q3O-(Glc)-Gal (Table S1) was energy optimised with the program Avogadro 1.2.0 (Hanwell et al., 2012). For Cor a 1.0501 the amino acid residues 23, 24, 31, 35, 38, 57, 59, 61, 63, 68, 70, 82, 84, 101, 117, 121, 137, 138, 140, 144, were used. For Cor a 1.0401 the residues 141, 145, 84, 101, 103, 70, 86, 56, 36, 40, 42, 59, 37, 138, 139, 31, 142, 24, 23, 57, 116, 105, 82, 118 were selected as active residues, as these form the binding pocket and its entrance. For distance restraints used for HADDOCK (Table S2), the carbon atoms of the corresponding amino acid residues and the corresponding protons of the ligands derived from the NOESY experiments were used and set between 5 and 8 Å. For clustering the RMSD value was used with a cutoff of 2.5 Ǻ.

## 3 Results and discussion

### 3.1 Identification of potential Cor a 1 ligands by mass spectrometry

We have recently shown that there exist a lot more Cor a 1 isoallergens and variants than previously known (Hendrich et al., 2024). So far eight Cor a 1 isoallergens have been identified which could be detected in different plant tissues (Breiteneder et al., 1993; Hendrich et al., 2024; Lüttkopf et al., 2002). In addition, the glycosylated flavonoid Q3O-(Glc)-Gal was found to be a natural ligand for the isoallergen Cor a 1.0401 (Jacob et al., 2019). Moreover, it has been demonstrated that the binding of flavonoids to Bet v 1.01 is variant dependent (Seutter von Loetzen et al., 2015). Bound ligands are thought to stabilize allergens and to enhance the immune reaction (Chruszcz et al., 2021). Therefore, identifying potential interaction partners for isoallergens is crucial for understanding and potentially mitigating the allergenic properties of Bet v 1 and Cor a 1.

**Fig. 1:**
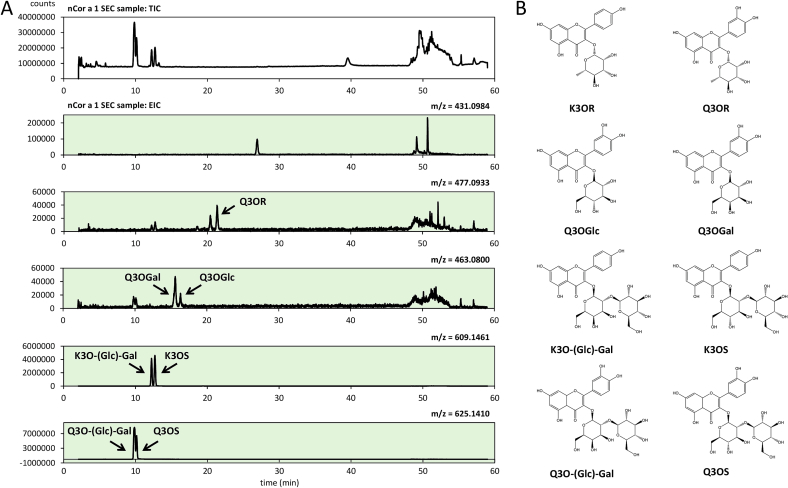
**UHPLC/QTOF-MS. A)** Total ion count (TIC) chromatogram of nCor a 1 after SEC and extracted ion count of ligand specific calculated mass (green background). **B)** Corresponding molecular structure of identified ligands. (For interpretation of the references to colour in this figure legend, the reader is referred to the web version of this article.)

To further elucidate the binding properties of Cor a 1 isoallergens to glycosylated flavonoids, we set out to identify new ligands and characterise their binding specificities. Thus, natural (n)Cor a 1, which is in principle a mixture of different ligand-bound Cor a 1 isoallergens, was isolated from hazel pollen extract and analysed by UHPLC/QTOF-MS. For comparison, the same procedure was performed with hazel pollen extract, i.e. without prior nCor a 1 purification, which showed the presence of the same potential ligands. Among other substances, such as caffeoyl-spermidine (data not shown), several glycosylated flavonoid derivatives were identified (Fig. 1; Fig. S2, S3) and their binding properties further analysed. Determination of the accurate masses, as well as characteristic fragmentation patterns and chromatographic behaviour was used for identification are summarized in Table 1.

**Table 1:** Corresponding physical data from Fig. 1.

Compound	Formula	Calculated [M-H]^−^ (*m*/*z*)	Measured [M-H]^−^ (m/z)	Error (ppm)	Retention time (min)	Qualifier fragment ion (m/z)
Q3O-(Glc)-Gal	C_27_H_30_O_17_	625.14102	625.1429	3.007321	9.851	300.0287, 178.9987
Q3OS	C_27_H_30_O_17_	625.14102	625.1429	3.007321	10.149	445.0781, 300.0287, 178.9989
K3O-(Glc)-Gal	C_27_H_30_O_16_	609.14611	609.1457	−0.673073	12.240	429.0853, 284.0350
K3OS	C_27_H_30_O_16_	609.14611	609.1457	−0.673073	12.714	429.0834, 284.0336, 178.9992
Q3OGal	C_21_H_20_O_12_	463.0882	463.0888	1.295650	15.604	300.0276
Q3OGlc	C_21_H_20_O_12_	463.0882	463.0893	2.375357	16.332	300.0284
Q3OR	C_21_H_20_O_11_	447.0933	447.0935	0.447334	21.332	300.0286
K3OR	C_21_H_20_O_10_	431.0984	431.0981	−0.695897	26.943	284.0325

To confirm the identity of the compounds the extracted ion count (EIC) for the specific mass [M^−^H]^−^ of the corresponding commercially available flavonoid derivatives was used. Q3O-(Glc)-Gal was purified as described (Jacob et al., 2019). K3O-(Glc)-Gal was not commercially available. Preparative purification from the pollen extract by reversed-phase HPLC yielded a mixture of the two compounds K3O-(Glc)-Gal and K3OS, which were clearly identifiable by NMR with a ^1^H, ^13^C HSQC (Fig. S4). Since separation of the two compounds failed, only K3OS was used in our studies. The monosaccharide flavonoid derivatives K3OR, Q3OR and the disaccharide flavonoid derivatives K3OS, as well as the already known binding ligands (Jacob et al., 2019; Seutter von Loetzen et al., 2014; Strack et al., 1984) Q3OS and Q3O-(Glc)-Gal were clearly identifiable (Fig. 1, Table 1; Fig. S2, S3).

The EIC and fragmentation patterns (Fig. 1, Fig. S2, S3) indicated the presence of Q3OGlc (*m*/*z* = 463.08) or quercetin-3-O-β-D-galactopyranoside (Q3OGal) (m/z = 463.08), since a quercetin fragment (m/z = 300.02) is formed during the fragmentation. Since glucose and galactose have identical masses, resulting in the same m/z of glycosylated quercetin, they cannot be distinguished based on their spectra. However, additional analyses with Q3OGlc, Q3OGal, and the hazel pollen extract using a Vanquish HPLC coupled to an Orbitrap confirmed the presence of Q3OGal in the extract (Fig. S3-F1, S3-F2). In addition, Q3OGlc was previously shown to be present in male hazel flowers and nut (Cerulli et al., 2018; Masullo et al., 2016).

### 3.2 Chemical shift assignment of Cor a 1.0501 and titrations with potential ligands

Structural changes induced by the binding of polyphenols/flavonoids can alter the allergenic potential and modulate the immune response as has been shown in previous (model) studies for allergenicity e.g. of the birch pollen allergen Bet v 1, peanuts or milk proteins (Chruszcz et al., 2021; Plundrich et al., 2017; Wu et al., 2018). Therefore, binding studies are necessary for understanding allergenicity and potentially provide a basis for reducing the allergenicity of foods.

Circular dichroism (CD) melting curves performed previously exhibited that Cor a 1.0501 is a very stable protein with a high melting point (T_m_ = 84.32 °C) (Hendrich et al., 2024). Thus, this isoallergen was chosen for backbone assignment by NMR analysis followed by titrations with the putative ligands. An NMR spectrum correlating resonance frequencies of amide protons and directly bonded ^15^N-labelled nitrogen atoms (2D ^1^H,^15^N HSQC, heteronuclear single quantum correlation) allows the individual detection of peptide backbone amide signals. Since each signal represents a single amino acid of the peptide chain, such a spectrum provides information about the folding state of a protein. The position of each signal in the spectrum depends on the chemical environment, which is different for each amino acid in a folded protein. The corresponding ^1^H, ^15^N HSQC spectrum (Fig. S5) of Cor a 1.0501 shows that the residues are well dispersed (6.9–9.7 ppm) indicating a folded protein with a defined structure. 93 % of the residues could be assigned (Fig. S5A, B). Formation of secondary structures in proteins causes systematic deviations of backbone chemical shifts from random coil values in folded proteins. The direction of deviation of the chemical shifts relative to the random coil values is expressed as Chemical Shift Index (CSI) and allows the determination of sequence specific secondary structure elements. Upfield chemical shift changes (lower resonance frequencies, lower ppm values) of the α-protons (HA; negative CSI values) and downfield chemical shift changes for the α- and carbonyl carbons (CA, CO, positive CSI value) can be observed for helical secondary structures, whereas β-strands show the opposite direction of chemical shifts The CSI values of Cor a 1.0501 show secondary structure elements whose arrangement corresponds well to that of other PR-10-like proteins (Fig. S5C). Furthermore, a 3D model of Cor a 1.0501 was created with AlphaFold 2 (Jumper et al., 2021) (Fig. S5D). The model structure obtained is consistent with known structures of PR-10 proteins (Chruszcz et al., 2021; Fernandes et al., 2013; Gajhede et al., 1996; Jacob et al., 2019; Seutter von Loetzen et al., 2012; Seutter von Loetzen et al., 2014). The amphiphilic cavity formed by the structural elements is large enough to accommodate small ligands such as glycosylated flavonoids. (Jacob et al., 2019).

**Fig. 2:**
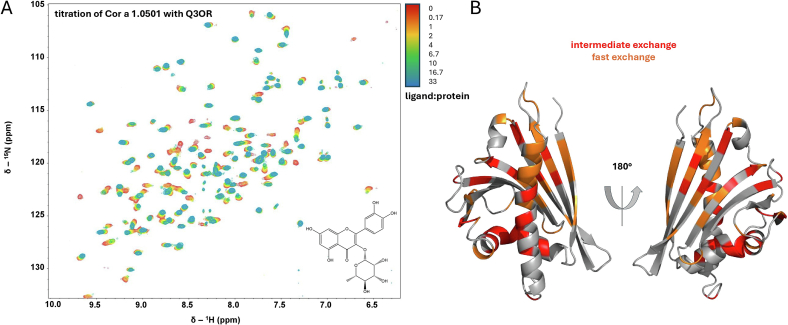
**Binding of Cor a 1.0501 to Q3OR. A)**^1^H, ^15^N HSQC spectrum of the titration of ^15^N-labelled Cor a 1.0501 with Q3OR. Colour coding indicates the ligand:protein ratio as shown by the bar at the bottom right of the ^1^H, ^15^N HSQC spectrum. Measurement was performed with 60 μM ^15^N-Cor a 1.0501 at 298 K in 10 mM Na_2_HPO_4_/NaH_2_PO_4_ buffer, 50 mM NaCl at pH 7.5, 10 % ^2^H_2_O with Bruker Avance 900 MHz spectrometer. (The titrations of the other ligands are shown in the supplement in Fig. S6)·**B)** 3D Model of Cor a 1.0501 created with Alpha Fold 2. The residues that showed intermediate or fast exchange during the titration are highlighted in red and orange, respectively. (For interpretation of the references to colour in this figure legend, the reader is referred to the web version of this article.)

Titration of a ligand results in chemical shift changes of affected amino acids since ligand binding changes their chemical environment. The putative ligand Q3OR was titrated to Cor a 1.0501 and ^1^H,^15^N HSQC spectra were recorded for each titration step. An overlay of the ^1^H,^15^N HSQC spectra shows the chemical shift changes induced by binding of Q3OR (Fig. 2A).

To reveal the residues involved in ligand binding, the 3D model of Cor a 1.0501 created with AlphaFold2 (Fig. S5D) was used (Fig. 2B). The residues affected by ligand binding and showing intermediate or fast exchange in the NMR titration experiments are highlighted in red and orange, respectively (Fig. 2B).

Titrations with the monosaccharide derivatives K3OR, Q3OGal, and Q3OGlc, as well as the disaccharide derivatives Q3OS, K3OS and Q3O-(Glc)-Gal were performed (Fig. S6). While the monosaccharide flavonoids showed binding, none of the disaccharide derivatives tested bound to Cor a 1.0501. Moreover, the binding affinities of the monosaccharide flavonoids were determined by measuring the K_D_-values of several amino acid residues undergoing fast exchange during ligand titration. The average values of these measurements for each ligand were in a range of 86 μM to 348 μM (Fig. S6). Since only the amino acid residues being in the fast exchange can be measured, less accurate and usually higher K_D_-values are expected than if a method that considers the overall binding affinity is used.

To confirm ligand binding, isothermal titration calorimetry (ITC) was carried out (Fig. S7). Although this method is not ideal for K_D_-values above 100 μM, it was used because fluorescence titrations could not be performed due to the lack of tryptophan in the protein. Measurements were performed using the same pH conditions as for the NMR experiments. The K_D_-values determined with the four compounds, Q3OR, K3OR, Q3OGlc and Q3OGal, are all quite similar, ranging from ca. 70 to 170 μM and comparable to the average values obtained by NMR (Fig. S6, S7).

### 3.3 Ligand screening with Cor a 1 isoallergens

Previous studies have shown that the isoallergen Cor a 1.0401 binds of Q3O-(Glc)-Gal specifically (Jacob et al., 2019). To gain deeper insight into the binding specificities of Cor a 1 isoallergens, the ^15^N-labelled Cor a 1 isoallergens 1.0101, 1.0302, 1.0401, 1.0501, 1.0601, 1.0701 and 1.0801 were purified from *E. coli* as already described (Hendrich et al., 2024). Cor a 1.02 could not be expressed in a soluble form in *E. coli* (Hendrich et al., 2024) and could therefore not be included in the analyses. For initial screening experiments only one ligand concentration was added to 60 μM of a ^15^N -labelled isoallergen to find chemical shift events in the ^1^H, ^15^N HSQC spectra indicative of binding (Table 2, Fig. S8). Based on the results obtained above (see Figs. S6 and S7), a ligand excess of at least fivefold was chosen, as binding affinities in the range of approximately 100 μM to 200 μM were expected. According to the law of mass action, at a K_D_ of 100 μM, 75 % of the protein is in complex with the ligand, meaning that chemical shift changes should be visible.

**Table 2:**
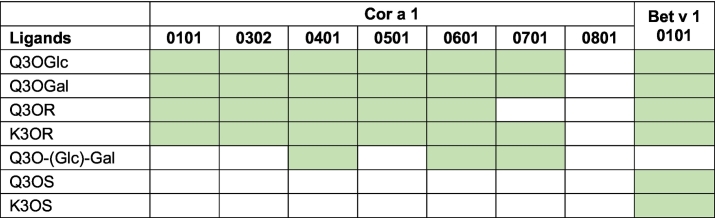
Summary of Cor a 1 isoallergens and ligand binding.

binding  no binding (at least 5-fold excess of ligand).

Data are based on HSQC screening (Fig. S7) and are consistent with HSQC titrations (Fig. S6) and ITC experiments (Fig. S7).

The ^1^H, ^15^N HSQC spectra revealed binding of flavonoid monosaccharide derivatives by all Cor a 1 isoallergens except Cor a 1.0801. However, this is not surprising, as the potential ligands were isolated from pollen and Cor a 1.0801 was shown to be present only in immature nuts, and not in pollen (Hendrich et al., 2024). All the other isoallergens bound to Q3OGlc, Q3OGal, and K3OR. Only Cor a 1.0701 was not able to bind Q3OR (Table 2, Fig. S8). The flavonoid disaccharides exhibited greater differences in binding, indicating a higher specificity: Binding of Q3O-(Glc)-Gal to Cor a 1.0401 has previously been shown (Jacob et al., 2019). In addition, it was also bound by Cor a 1.0601 and 1.0701. These isoallergens are weakly expressed in hazel pollen (Hendrich et al., 2024).

None of the Cor a 1 isoallergens bound to the flavonoid sophorosides Q3OS and K3OS (Table 2; Fig. S8). In contrast, these compounds were bound by Bet v 1.0101 from birch pollen, which was used as a control (Table 2; Fig. S8). As already known, Bet v 1 binds specifically to the sophoroside Q3OS but not to Q3O-(Glc)-Gal. (Jacob et al., 2019).

To further investigate the binding properties and to identify specific residues responsible for ligand binding Cor a 1.0501 was selected for further analysis because we had shown in a previous publication that it is a particularly stable protein (Hendrich et al., 2024), with clear, easily assigned signals in the HSQC spectrum (Fig. S5).

### 3.4 Isotope edited/filtered nuclear Overhauser enhancement spectroscopy

To gain a better understanding of the different binding behaviours of mono- and diglycosylated flavonoids, further NMR binding studies were conducted. Although chemical shift changes in the protein can be used to show which amino acid residues are affected by ligand binding, this information was not sufficient to determine the orientation of the ligand by modelling. Since the ligands bind in the amphiphilic pocket of the protein, many chemical shift changes are due to general conformational changes of the protein and thus the affected amino acid residues do not directly interact with the ligand.

Thus, to obtain a complex structure of Cor a 1.0501 and to demonstrate the orientation of a ligand in the amphiphilic cavity, isotope edited/filtered NOESY experiments were performed with Q3OGlc. Intermolecular NOE interactions are used for characterizing interfaces. For two spatially close protons (distance less than 5 Ǻ) an NOE interaction can be detected. In an isotope filtered/edited NOESY experiment the magnetic transfer of a ^13^C bound proton of the protein to a ^12^C bound proton of the ligand can be observed and gives information about the interaction of the protein with the ligand.

**Fig. 3:**
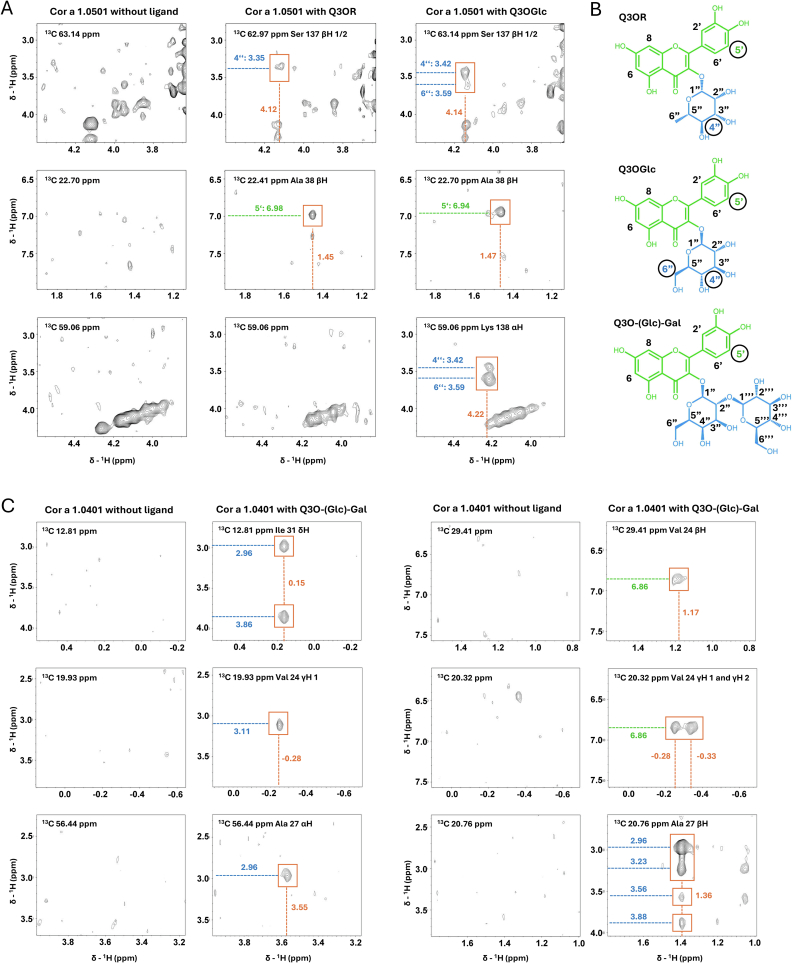
**Filtered/edited NOESYs of Cor a 1.0501 in complex with Q3OR or Q3OGlc, and Cor a 1.0401 in complex with Q3O-(Glc)-Gal. A)** Filtered/edited NOESY of Cor a 1.0501 without ligand (left column), with Q3OR (middle column), and with Q3OGlc (right column). Spectra show a cross section in the corresponding ^13^C level. NOE signals caused by the ligands are marked by orange boxes. The green and blue lines show the ^1^H signals caused by the quercetin and sugar moieties of the ligand. The orange lines show the ^1^H signals caused by the amino acid, respectively. **B)** Molecular structures of Q3OR, Q3OGlc and Q3O-(Glc)-Gal. The green and circled numbers represent the protons that build up the NOE with the protein (green and blue lines in A and C). **C)** Filtered/edited NOESY Cor a 1.0401 without ligand (left column), with Q3O-(Glc)-Gal (right column). Spectra show a cross section in the corresponding ^13^C level. NOE signals caused by the ligands are marked by orange boxes. The green and blue lines show the ^1^H signals caused by the quercetin and sugar moiety of the ligand. The orange lines show the ^1^H signals caused by the amino acid, respectively. (For interpretation of the references to colour in this figure legend, the reader is referred to the web version of this article.)

Using ^13^C-labelled Cor a 1.0501 in complex with the ligand Q3OR two NOE signals, and with the ligand Q3OGlc, three NOE signals could be assigned to the ligand and to amino acid side chains of the protein (Fig. 3; Fig. S9). The aromatic 5′ protons of Q3OR and of Q3OGlc show an NOE signal to the β protons of Ala 38, whereas the 4″ protons of the sugar moiety of Q3OR and Q3OGlc show an NOE signal with the β proton of Ser 137. For Q3OGlc, also the 6″ proton of the sugar moiety forms an NOE signal with the α proton of Lys138. In addition, ^1^H, ^13^C HSQC spectra of the complex were performed to confirm the signals derived from the ligand (Fig. S10).

**Fig. 4:**
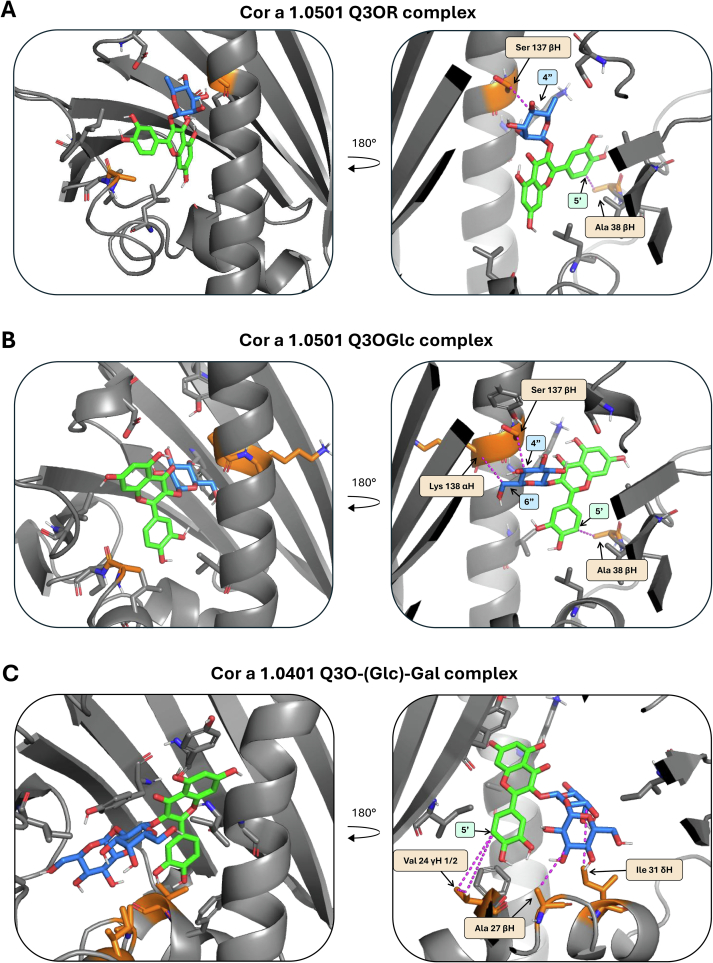
Docking and Complex Structures. Complex of Cor a 1.0501 with Q3OR **(A)** and Q3OGlc **(B)** and Cor a 1.0401-3CS with Q3O-(Glc)-Gal **(C)**. The left side shows the entrance to the binding pocket and the right side shows the binding from the opposite side. The quercetin moiety is highlighted in green and the sugar moiety in blue. The orange amino acids form a NOE signal to the ligand. The grey amino acids are located in close proximity to the ligand. Magenta coloured lines connect the carbon atoms used for distance restraints. (For interpretation of the references to colour in this figure legend, the reader is referred to the web version of this article.)

This distance information of the NOESYs was then used to define distance restraints for docking the ligands Q3OR and Q3OGlc into the model structure (Fig. 4A, B) of Cor a 1.0501 using the HADDOCK 2.4 webserver (Honorato et al., 2021, Honorato et al., 2024). The structure models of the Cor a 1.0501/Q3OR and Cor a 1.0501/Q3OGlc complexes (Fig. 4A, B) represent the interactions obtained by the NOE signals. The data show that the 3,4-dihydroxyphenyl ring of the quercetin moiety (labelled in green, Fig. 4A, B) of Q3OR and Q3OGlc is located at the entrance of the cavity with Ala 38 fixing the 5′ proton of the 3,4-dihydroxyphenyl ring, whereas the sugar moiety (labelled in blue, Fig. 4A, B) points into the cavity, and its 4″ protons interact with Ser 137 (Fig. 4A, B).

Since it was suggested that the sugar moieties of the flavonoid derivatives may be important for binding specificity (Chruszcz et al., 2021), and the NOESY and docking experiments indicated that Ser 137 is relevant for sugar binding, we mutated the corresponding residues in Cor a 1.0401 (G138S) and Bet v 1.0101 (S137A) (Fig. S11), as these two proteins exhibit different binding preferences for Q3O-(Glc)-Gal, which binds only to Cor a 1.0401, and Q3OS, which binds only to Bet v 1.0101 (Jacob et al., 2019). However, HSQC titrations with the ligands showed that Cor a 1.0401-G138S and Bet v 1-S137A were still able to bind to their ligands (Fig. S12).

In addition, docking experiments were performed with Cor a 1.0501 and Q3O-(Glc)-Gal, which was not bound by the protein in the experiments shown above, to determine the cause of this observation. Using binding of Ser 137 to the galactose moiety as a restraint, Q3O-(Glc)-Gal could not dock to Cor a 1.0501 in the same position and orientation as the monosaccharide derivatives Q3OR or Q3OGlc, suggesting that this is sterically impossible.

Since in contrast, Cor a 1.0401 and Bet v 1.0101 bind disaccharide flavonoid derivatives (Jacob et al., 2019; Seutter von Loetzen et al., 2014), but did show no effect of the mutations introduced above to disrupt binding, we speculated that mono- and disaccharide derivatives might bind in different orientations. Thus, we used the partial amino acid side chain assignment of Cor a 1.0401 in complex with the disaccharide flavonoid derivative Q3O-(Glc)-Gal (Table S3) and performed isotope edited/filtered NOESY experiments to clarify whether differences in binding could be detected. Since Cor a 1.0401 is prone to dimerisation, the mutant protein Cor a 1.0401-3CS, in which three surface accessible cysteines were replaced by serines, was used for the measurements. Partial assignment of the protein in complex with Q3O-(Glc)-Gal was performed to identify interacting amino acid side chains.

The NOEs obtained indicate interaction of the three protons of the Val24 side chain (Val 24 β-proton, and Val 24 γ-protons 1/2) of the protein with the quercetin moiety, and of four protons of the amino acid side chains Val24, Ala27 and Ile31 (Val 24 γ-proton;, Ala 27 α-proton and β-proton; and Ile 31 δ-proton) with the disaccharide moiety of Q3O-(Glc)-Gal (Fig. 3C). These results strongly suggest a completely different binding mode for the disaccharide flavonoid, i.e. here the aromatic quercetin moiety is located deep in the cavity and the sugar moiety is situated at the entrance of the pocket (Fig. 4C).

## 4 Conclusion

Various glycosylated flavonoids could be identified by UHPLC/QTOF-MS and NMR analyses in pollen extracts and bound to nCor a 1, indicating that they are natural ligands. Screening analyses using ^1^H, ^15^N HSQCs revealed that Cor a 1 isoallergens exhibit different binding properties, suggesting different functions in the plant. Interestingly, Cor a 1.0801, which is not found in pollen but in the nut or flower does not bind any of the glycosylated flavonoids. Whereas Cor a 1.0501 was only able to bind to monosaccharide flavonoid derivatives, Cor a 1.0401 was also able to bind to disaccharide flavonoids. NMR NOESY experiments revealed that the sugar bound covalently to the flavonoid moiety is responsible for specific binding. Although Ser 137 located in the amphipathic cavity of the protein interacts with the 4″ protons of the sugar when monosaccharide flavonoids like Q3OR or Q3OGlc are used, this is not the case when Q3O-(Glc)-Gal is bound. Docking experiments based on the data obtained by NMR NOESY analyses revealed for the first time that the orientation of the monosaccharide flavonoids in the complex is completely different to that of the bound disaccharide flavonoids: The sugar moiety of the monosaccharide flavonoid protrudes into the amphiphilic cavity, whereas in the case of the disaccharide flavonoid the sugar moieties are located at the entrance of the pocket. These findings provide valuable information on how to determine ligand binding specificity for different isoallergens. In the long term, these results may provide an important basis for elucidating the different functions of isoallergens and their corresponding ligands in plants and thus may help to reduce the allergenic potential of foods.

## CRediT authorship contribution statement

**Julian M. Hendrich:** Writing – original draft, Visualization, Validation, Methodology, Investigation, Formal analysis. **Hatice Kara:** Validation, Investigation, Formal analysis. **Kristian Schweimer:** Writing – original draft, Formal analysis, Conceptualization. **Thessa P. Jacob:** Validation, Investigation, Formal analysis. **Maike Schneider:** Validation, Investigation, Formal analysis. **Susanne Baldermann:** Writing – original draft, Validation, Supervision, Formal analysis, Conceptualization. **Birgitta M. Wöhrl:** Writing – review & editing, Writing – original draft, Validation, Supervision, Project administration, Funding acquisition, Conceptualization.

## Declaration of competing interest

The authors declare that they have no known competing financial interests or personal relationships that could have appeared to influence the work reported in this paper.

## Data Availability

NMR data are available from the Biomolecular Magnetic Resonance Data Bank (BMRB): BMRB ID: 52769 (Cor a 1.0401-3CS), 52770 (Cor a 1.0501-Q3OR), 52771 (Cor a 1.0501-Q3OGlc), 52772 (Cor a 1.0501).

## Appendix A Supplementary data

Supplementary material Image 3
