# Supplementary material for: Identification of glycosylated flavonoids as new physiological ligands of the hazel allergen Cor a 1: Complex structures reveal different binding orientation and specificity of mono- and disaccharide derivatives
Source: Food Chem X. 2025 Jul 1;29:102711. doi: 10.1016/j.fochx.2025.102711 (PMC12275138; doi:10.1016/j.fochx.2025.102711)
Supplement: Supplementary file 1 — Supplementary material [file mmc1.docx]

Supplement

**Figures**

**
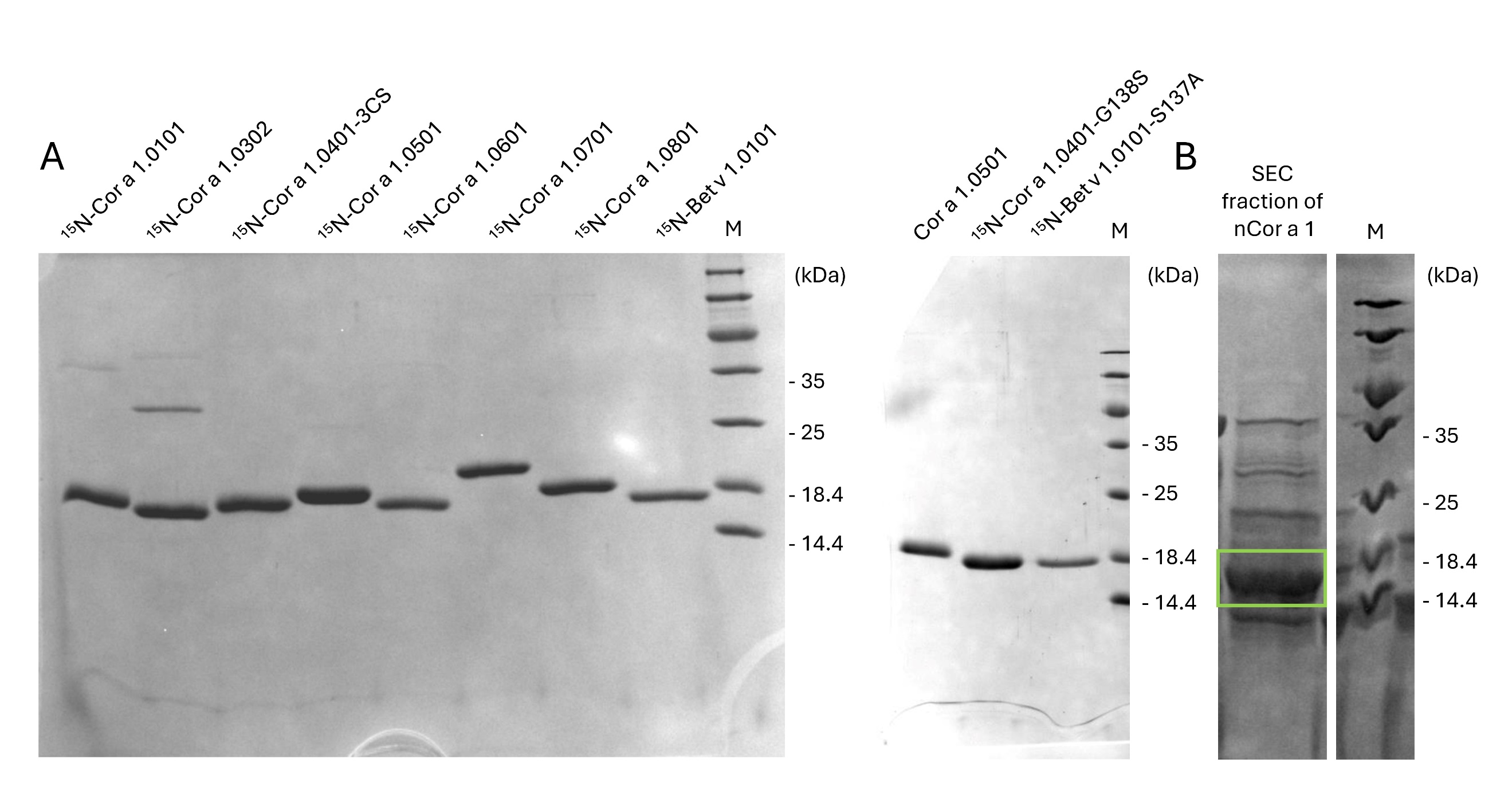
**

**Figure S1.** **SDS polyacrylamide gel electrophoresis of purified proteins**. **A)** Purified nonlabelled and labelled recombinant Cor a 1 and Bet v 1 proteins. **B)** Purified nCor a 1 used for fragmentation analysis. The band representing nCor a 1 is highlighted by a green box.

Lanes M, molecular weight standard; the apparent molecular weights (kDa) are indicated on the right.

**
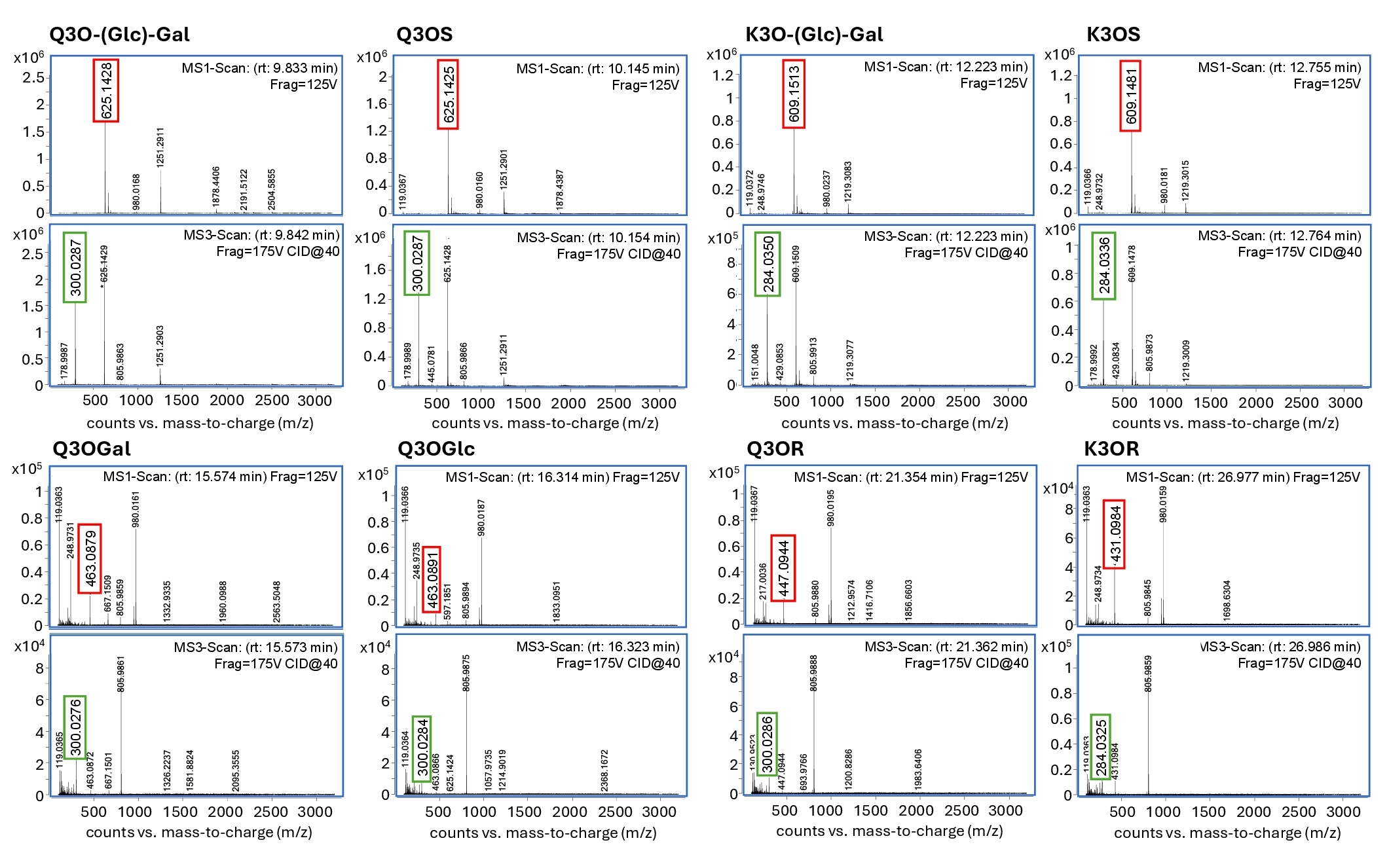
**

**Figure S2. Fragmentation analysis of the nCor a 1/ligand complex.** For each retention time of a ligand, the MS1 (upper MS spectrum) and the MS3 fragmentation (lower MS spectrum) are shown and refer to the EIC of purified nCor a 1 in Fig 1. For fragmentation a voltage of 175V and collision-induced dissociation (CID) of 40 eV was used. Red boxes: Mass of the corresponding ligand at its retention time. Green boxes: Quercetin or kaempferol after fragmentation of the ligand.

**
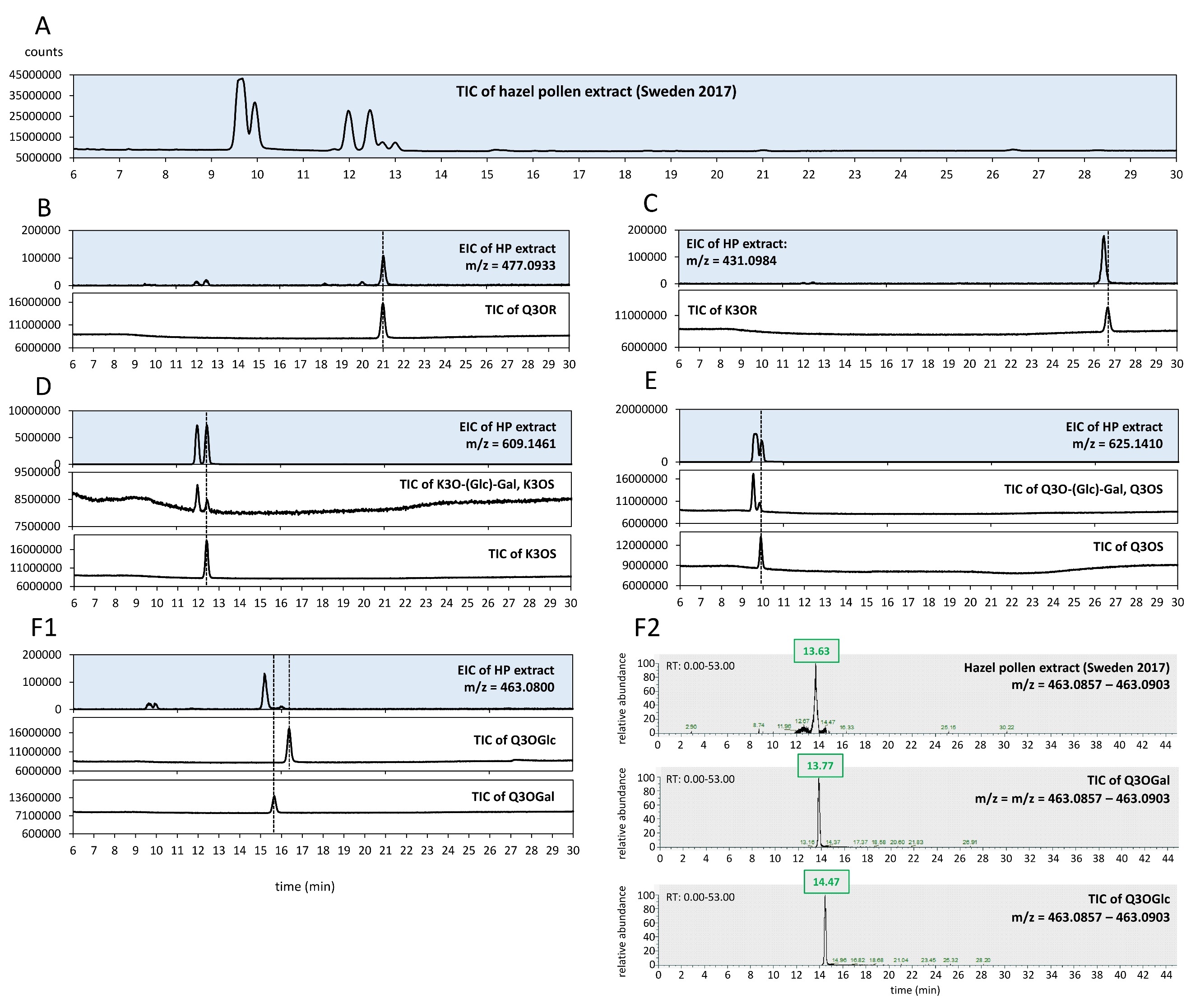
**

**Figure S3. Identification of flavonoids.** **A)** Chromatogram of the total ion count (TIC) of hazel pollen extract separated by UHPLC. **B)-F1)** Chromatograms of the extracted ion count (EIC) for the specific calculated mass [M-H]^-^ of the individual ligands from the hazel pollen extract (upper chromatograms) aligned with the TIC of the pure ligands (lower chromatograms). The pure ligands were similarly separated by UHPLC and used as a standard to identify the ligands in the hazel pollen extract. **D) and E)** The mixture of K3O-(Glc)-Gal/ K3OS and of Q3O-(Glc)-Gal/ Q3OS was purified form hazel pollen by preparative HPLC. K3OR, Q3OR, Q3OGlc, Q3OGal, K3OS and Q3OS were purchased. **F2)** For ligand identification, hazel pollen extract, Q3OGlc and Q3OGal were additionally measured on a Vanquish HPLC coupled to an Orbitrap Exploris 120 instrument.


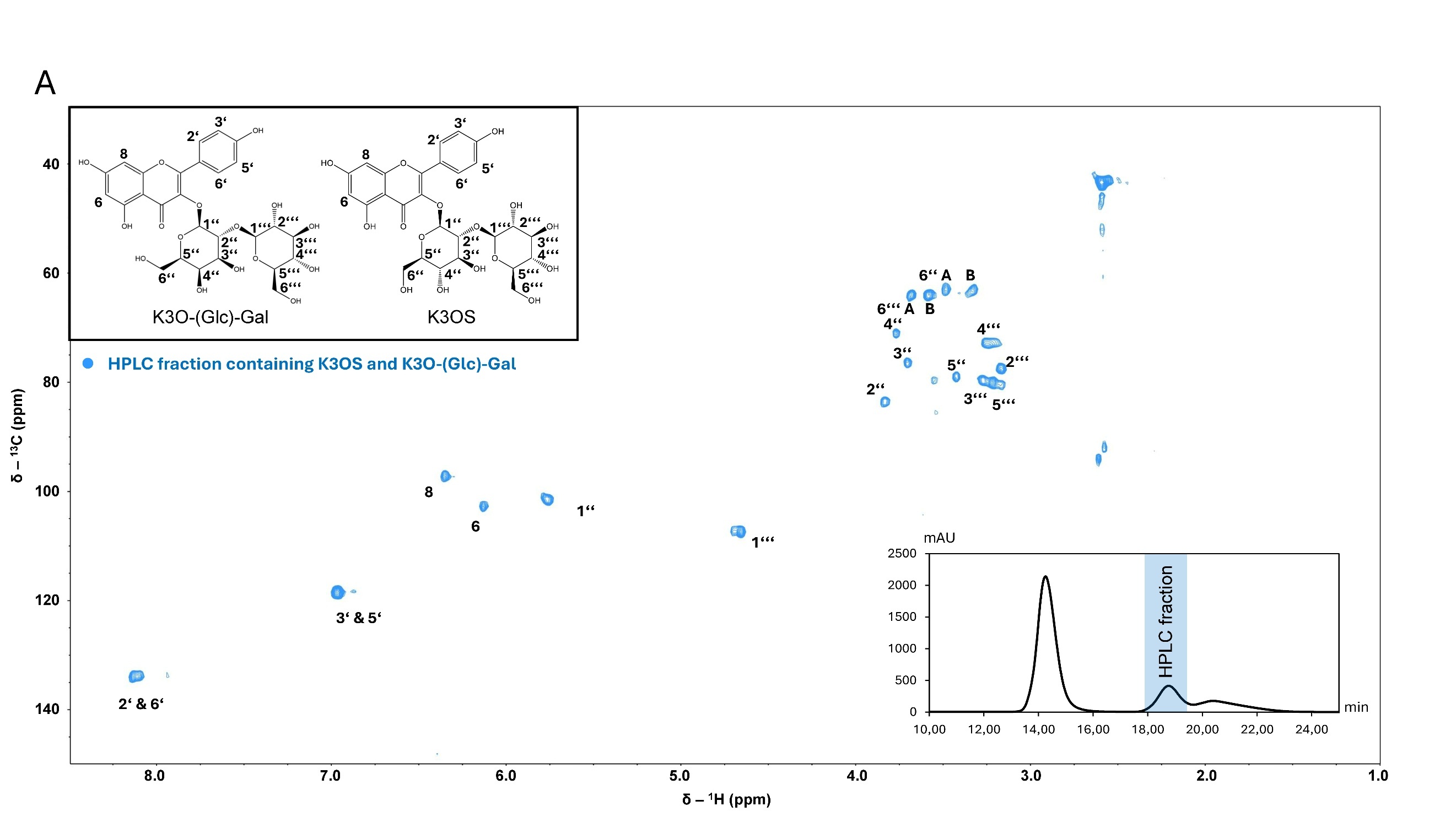


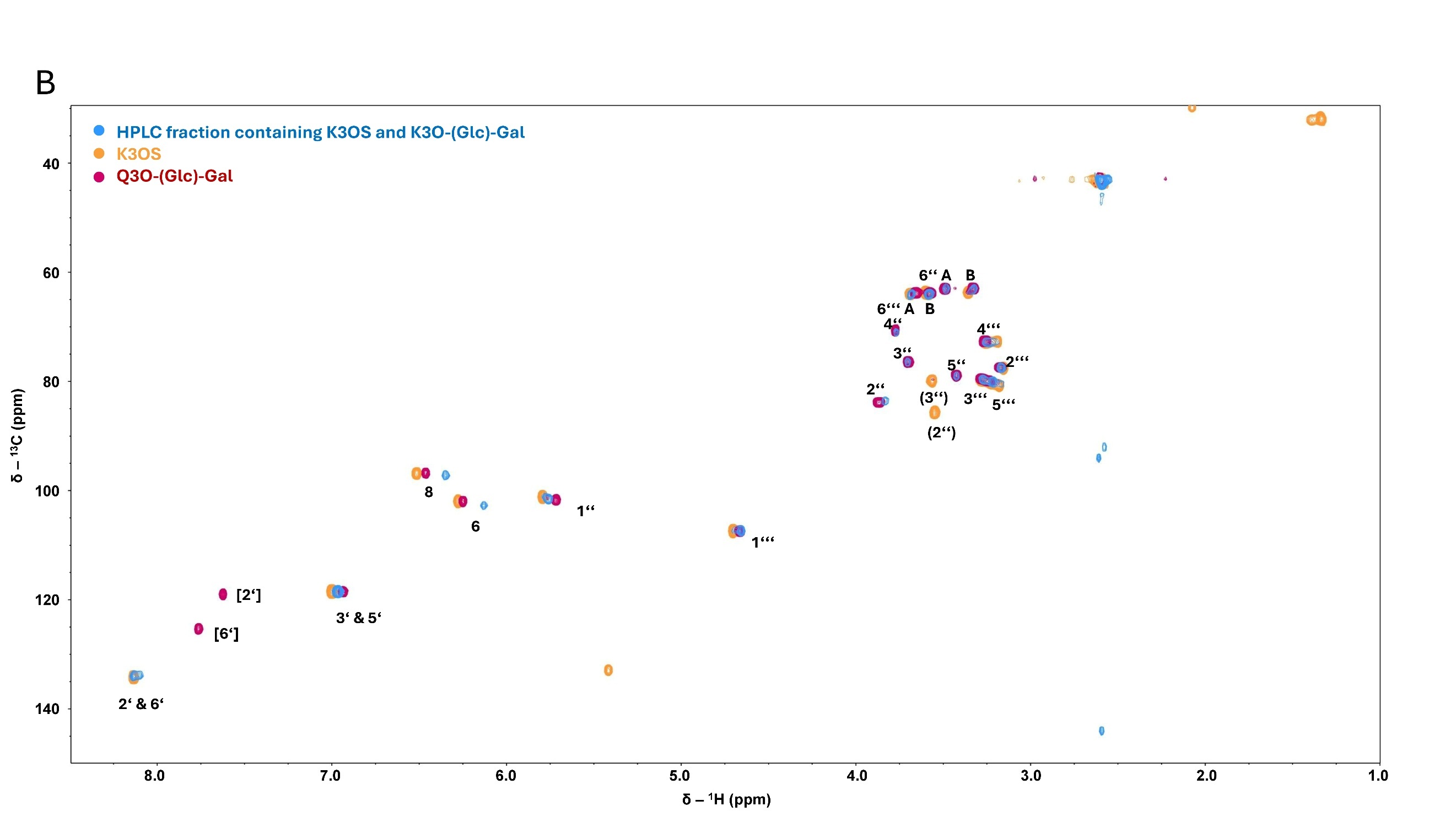


**Figure S4. ^1^H, ^13^C-HSQC NMR spectrum of a purified HPLC fraction containing K3OS and K3O-(Glc)-Gal.** The HPLC peak (blue box) was fractionated, lyophilised and resolved in d_6_DMSO. **A)** The ^1^H, ^13^C-HSQC of that HPLC fraction (blue signals) was measured at 298K on a 700 MHz spectrometer**. B)** K3OS and K3O-(Glc)-Gal were identified by superimposing these spectra with the spectra of purchased K3OS (orange signals) and Q3O-(Glc)-Gal (red signals) purified from hazel pollen. Numbers [2’] and [6’] mark the peaks of Q3O-(Glc)-Gal, numbers (3’’) and (2’’) refer to the peaks of K3OS.


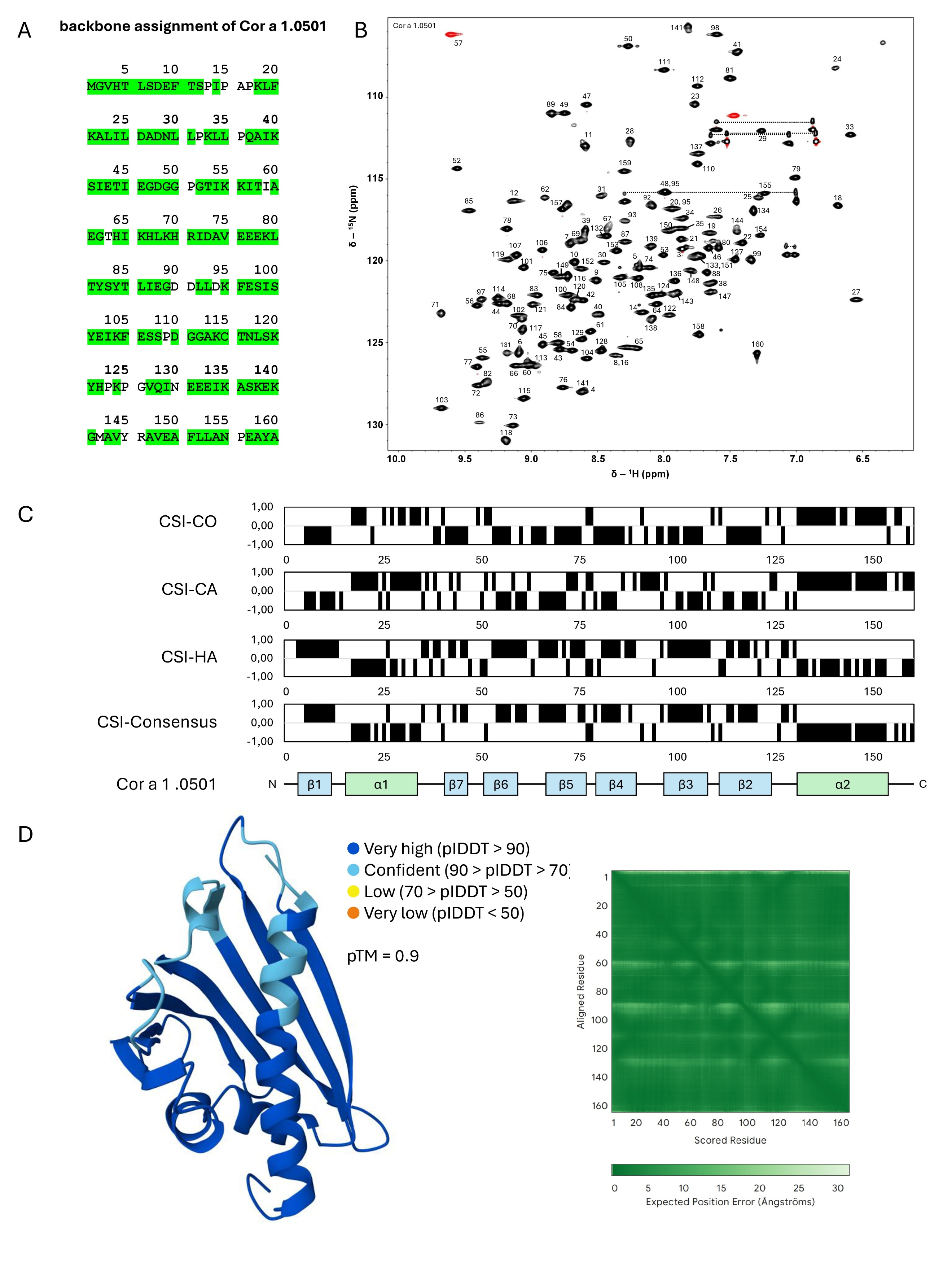


**Figure S5. Backbone assignment of Cor a 1.0501 and structure prediction.**  **A)** Amino acid sequence of Cor a 1.0501. Assigned residues are highlighted in green. **B)** ^1^H, ^15^N HSQC of 500 µM uniformly ^15^N-labelled Cor a 1.0501. Amide proton resonances are labelled according to their residue numbers. Measurement was performed at 298 K in 10 mM Na_2_HPO_4_/NaH_2_PO_4_ buffer at pH 7.5, 10% ^2^H_2_O, 1 mM DTT on a Bruker Avance 900 MHz spectrometer. **C)** Secondary chemical shift indices (CSI) for Hα, Cα, and CO nuclei, along with the consensus CSI combining all three nuclei. β-strands are represented by blue boxes and α-helical regions by green boxes. **D)** AlphaFold 2 structural prediction. Dark blue and light blue highlight regions with very high and high confidence, respectively, based on the predicted local Distance Difference Test (plDDT) scores.


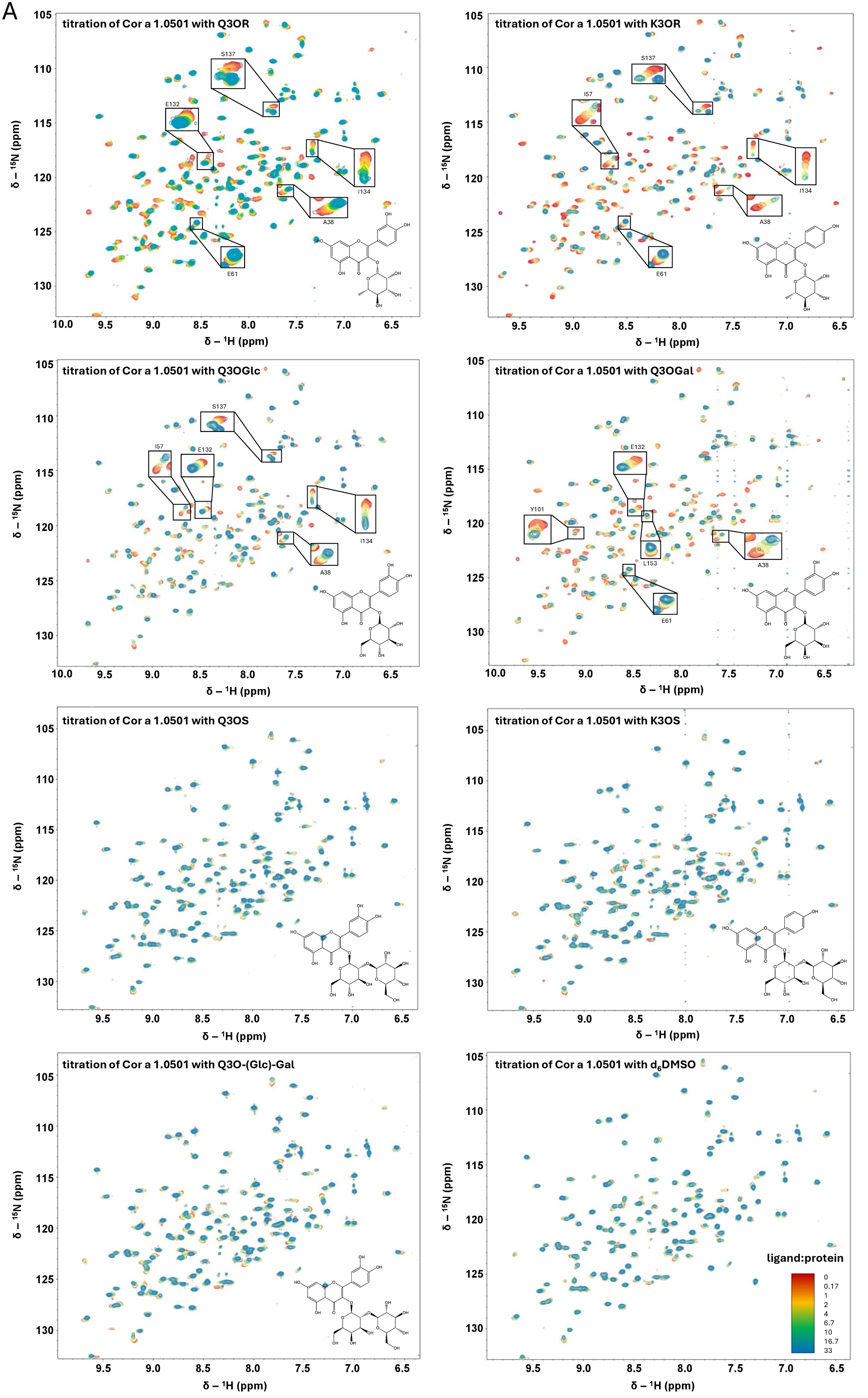


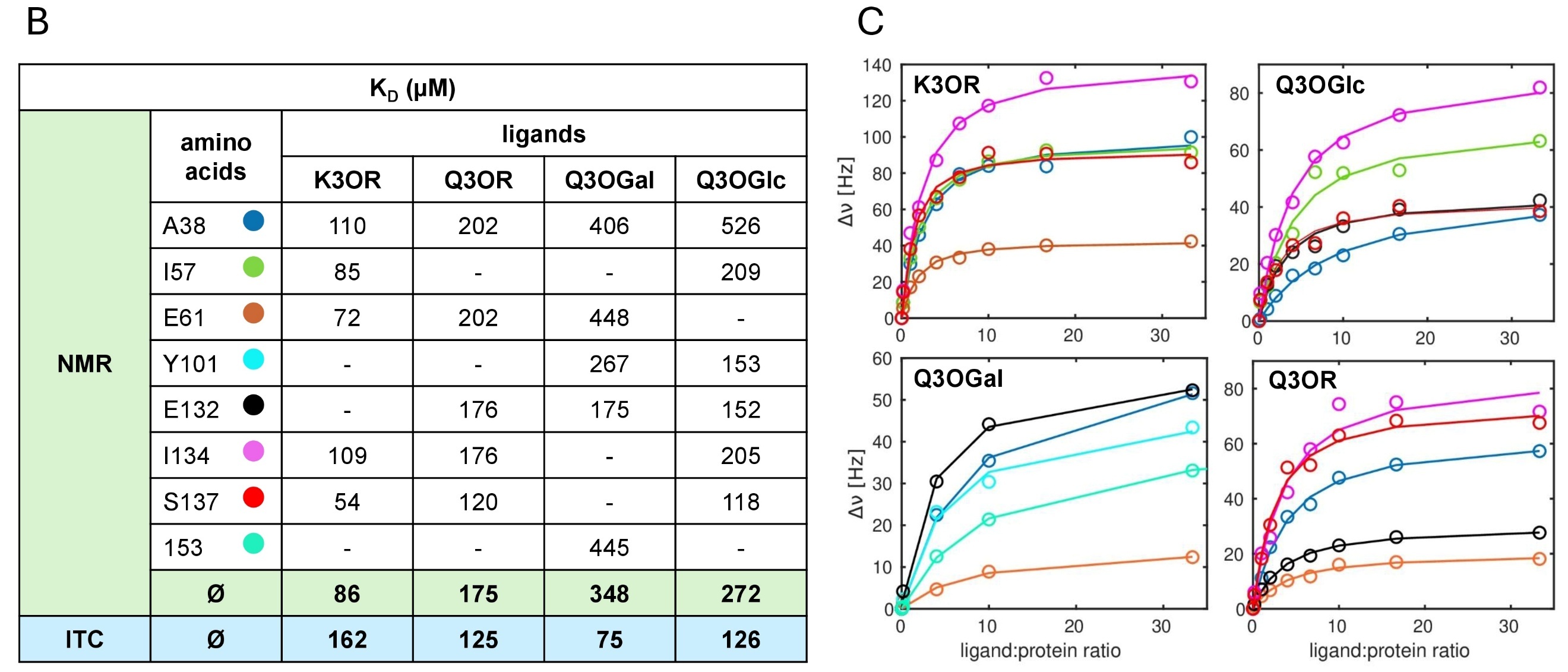


**Figure S6. Titration of Cor a 1.0501 with putative ligands. A)** ^1^H, ^15^N HSQC spectra of a titration of Cor a 1.0501 (60 µM) with the corresponding compounds dissolved in 100 (v/v) d_6_DMSO. A reference titration was performed using 100 % (v/v) d_6_DMSO. The boxes shown in the titration spectra with Q3OR, K3OR, Q3OGlc and Q3OGal show blow-ups of the chemical shift changes used for the determination K_D_-values. NMR buffer: 10 mM Na_2_HPO_4_/NaH_2_PO_4_ pH 7.5, 1 mM EDTA 50 mM NaCl and 10 % ^2^H_2_O. Data were recorded on a 900 MHz or 1GHz NMR spectrometer at 298K. **B)** Summary of K_D_-values for individual amino acid residues under fast exchange conditions determined by NMR titrations. Average K_D_-values of the amino acid residues analysed by NMR titrations for each protein are shown in green. For comparison the K_D_-values obtained by ITC measurements presented in Figure S7 are depicted in blue. Colour coding of the dots corresponds to the colours of the curves shown in C. **C)**. Fit of the curves to the data obtained by NMR titrations. Chemical shift perturbations (Δν) are plotted against the ligand:protein ratio for each ligand.


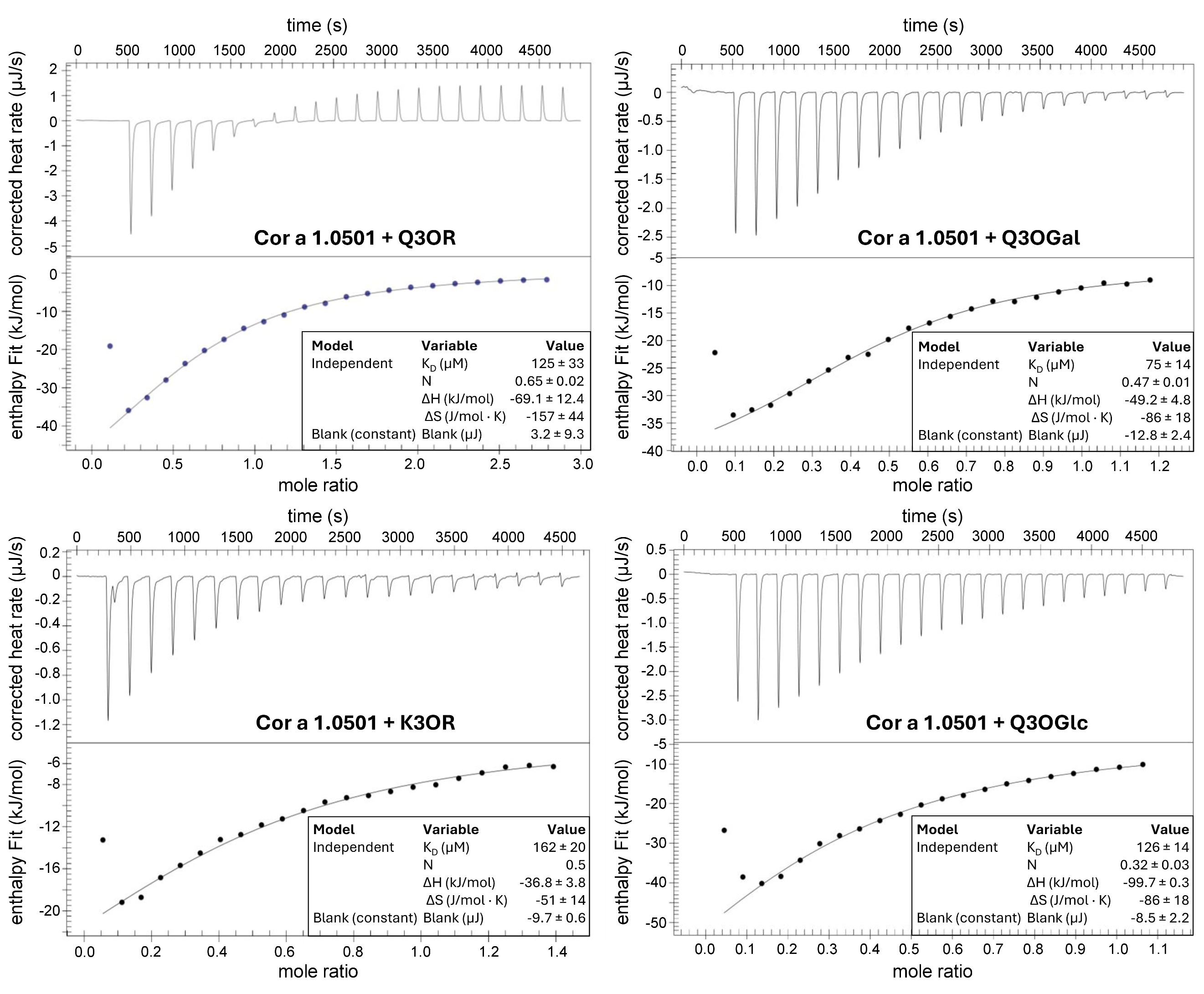


**Figure S7. Isothermal titration calorimetry.** Titration of Cor a 1.0501 (320 – 340 µM) with Q3OR (3.3 mM), K3OR (1.5 mM), Q3OGlc (1.3 mM) and Q3OGal (1.3 mM). Buffer: 10 mM Na_2_HPO_4_/NaH_2_PO_4_, 50 mM NaCl, pH 7.5 at 298 K. The heat rate was corrected with a buffer titration as a blank. Measurements were made in triplicate, with K3OR measurements were carried out in duplicate. Curves were fitted fitted to the data with an independent binding model using NanoAnalyze Data Analysis Version 4.0.0.4 (TA Instruments, USA). The first two data points were not used for fitting.


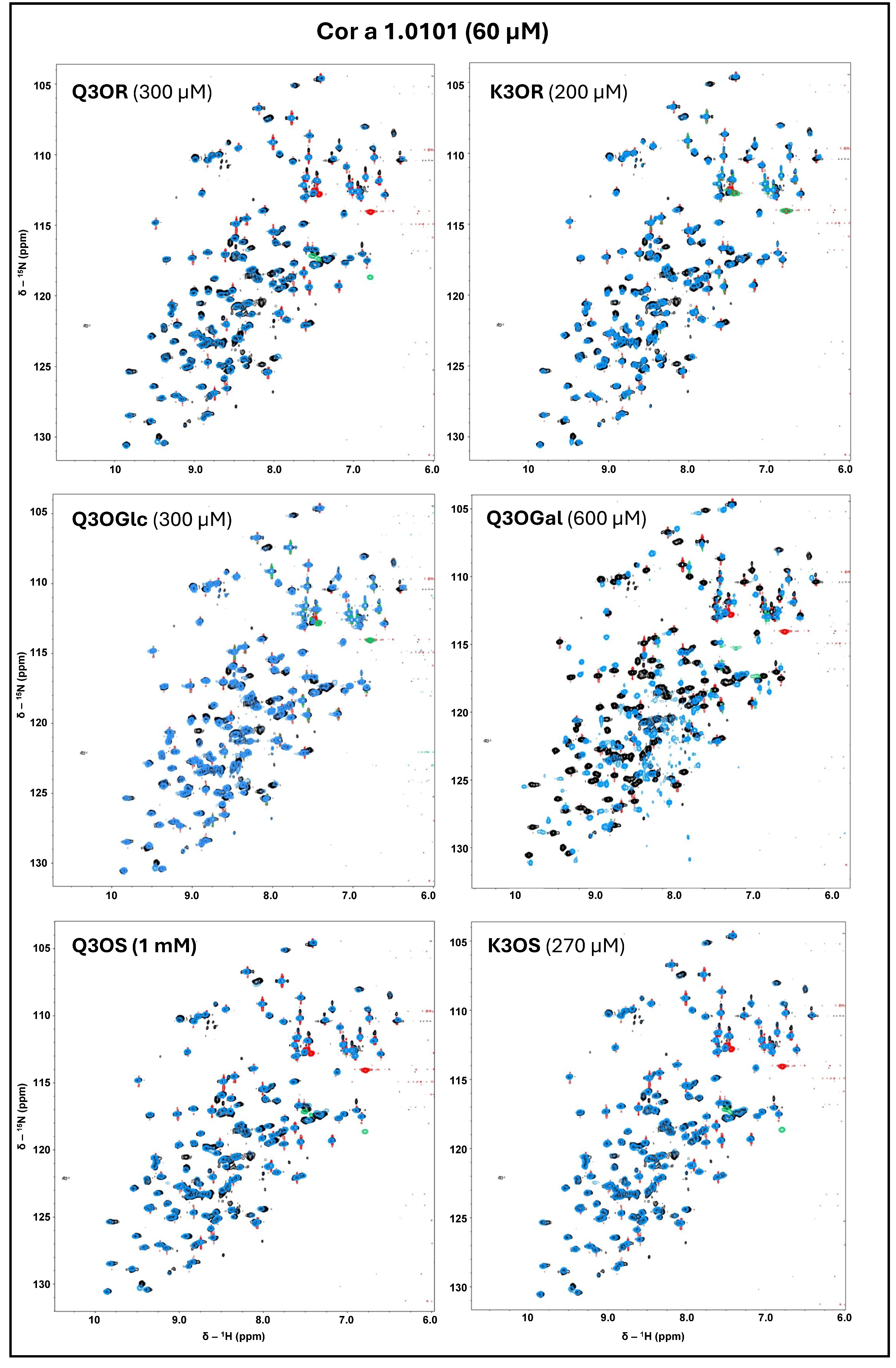


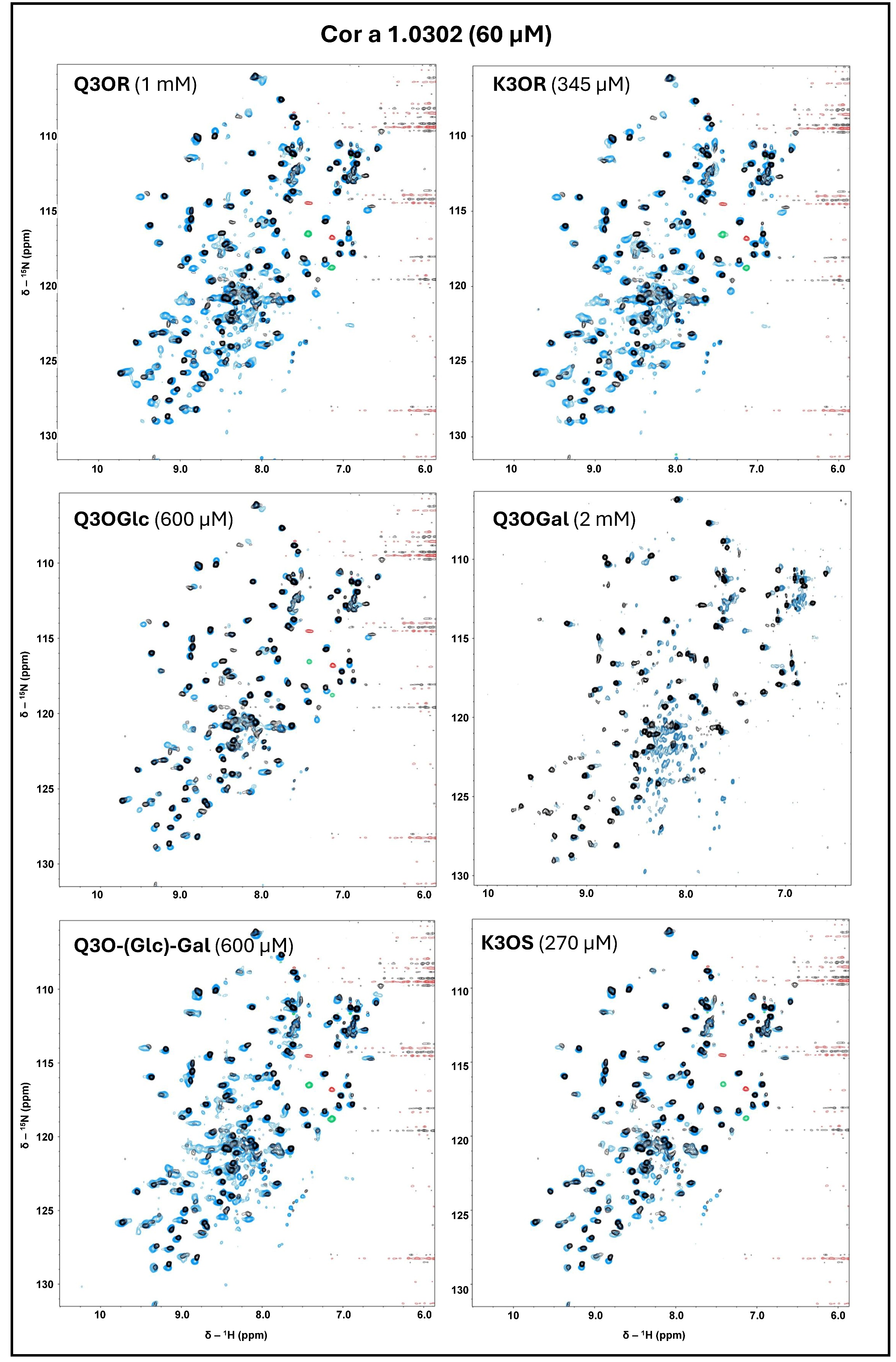


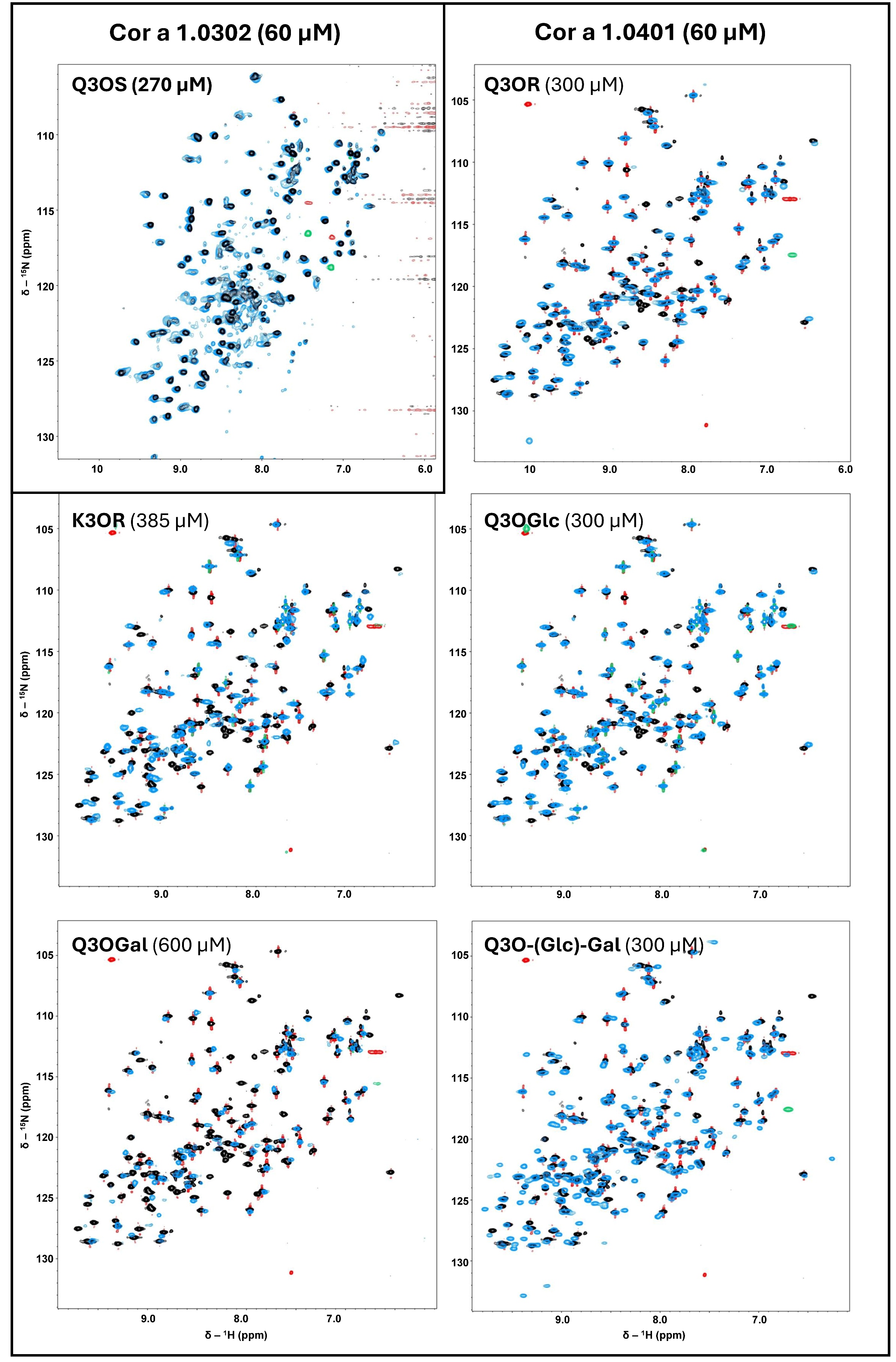


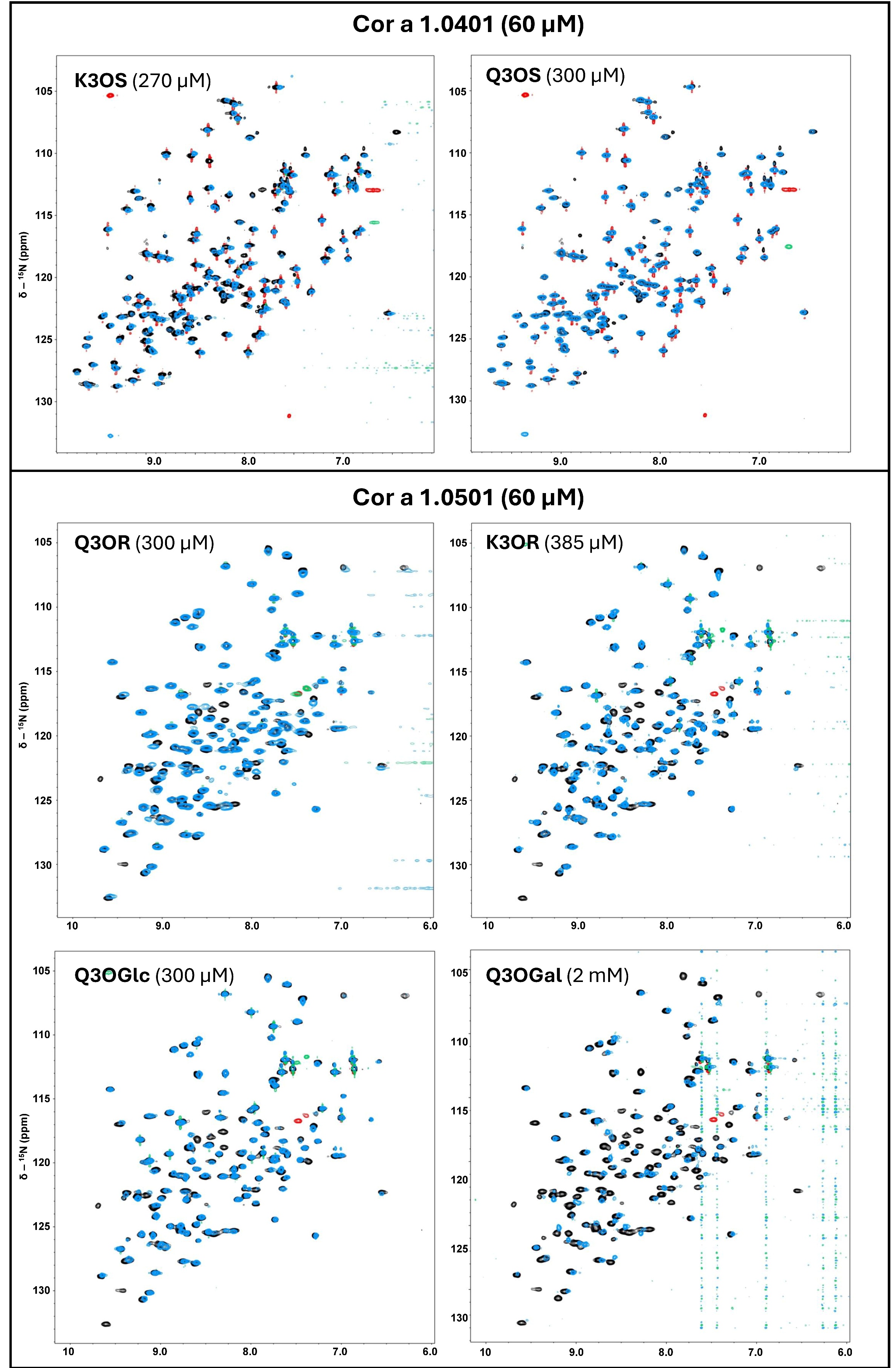


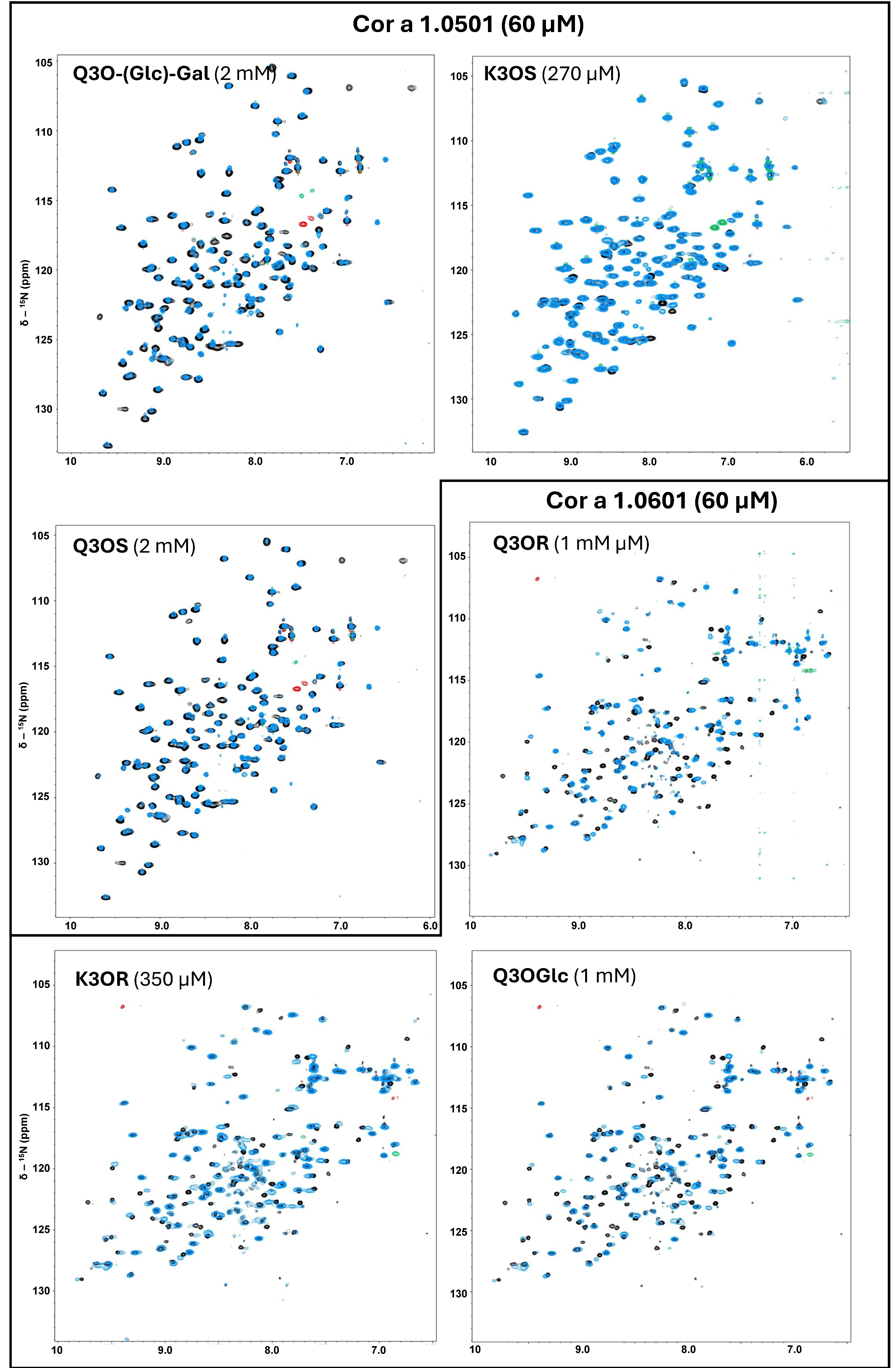


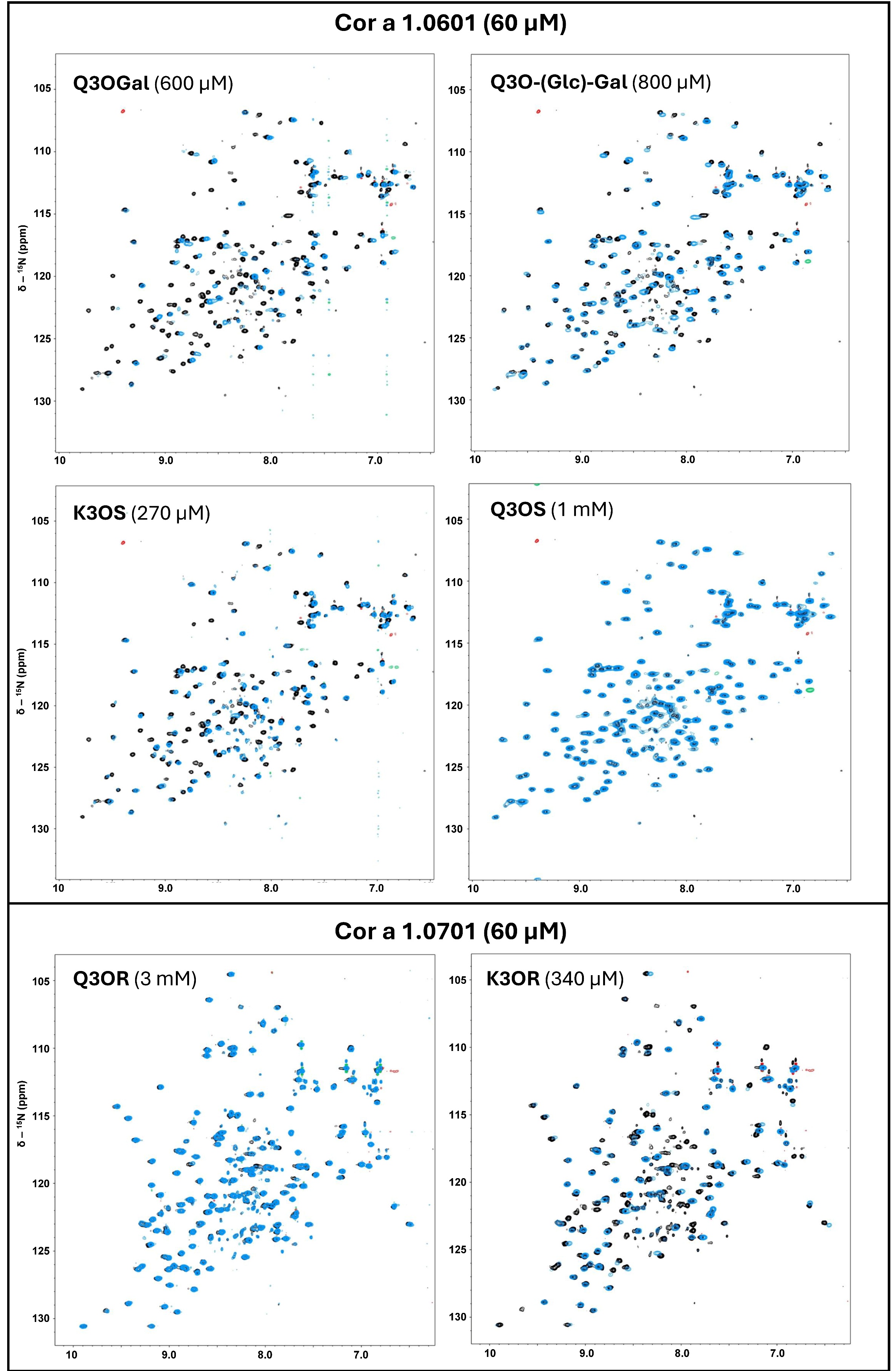


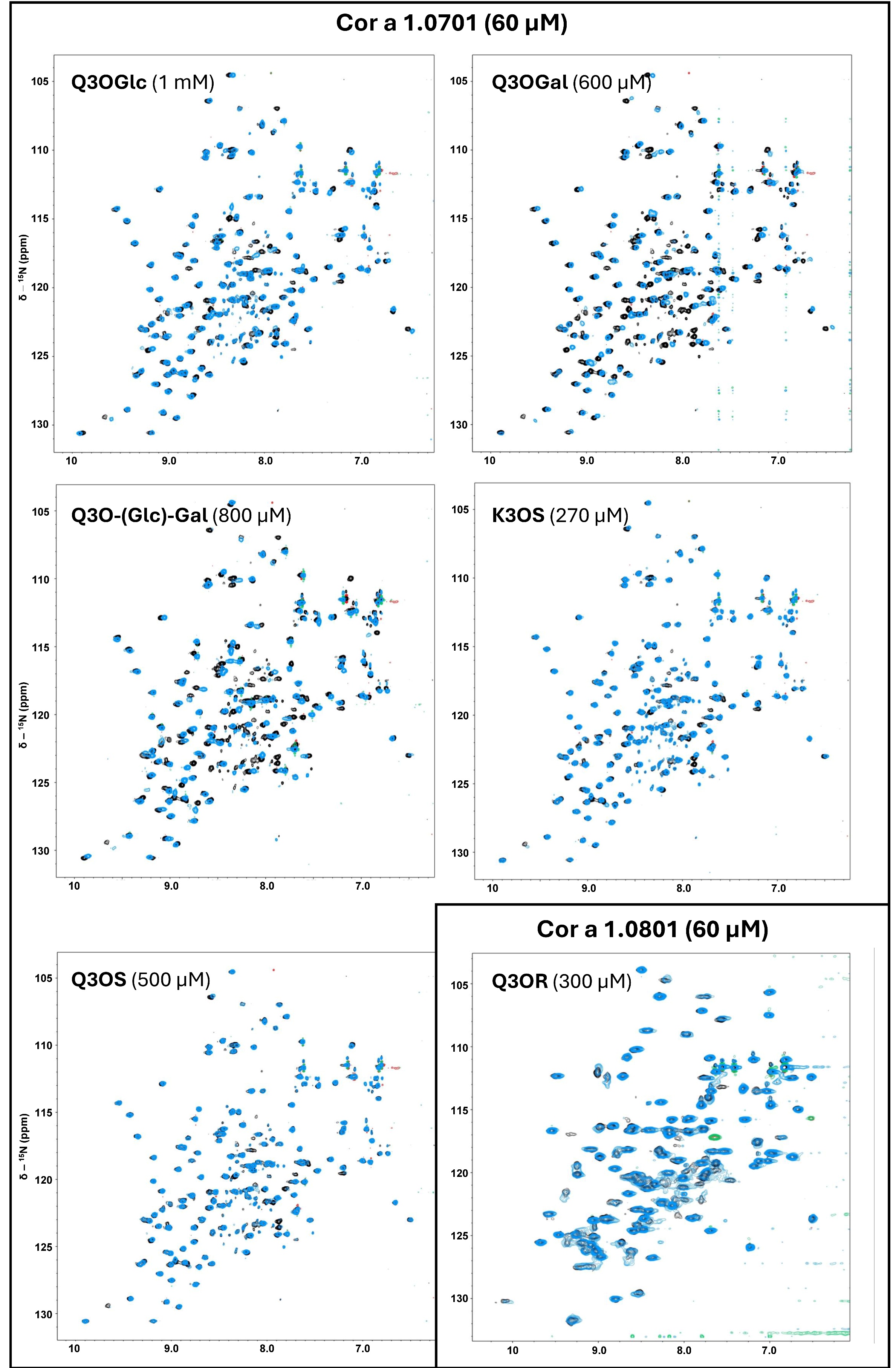


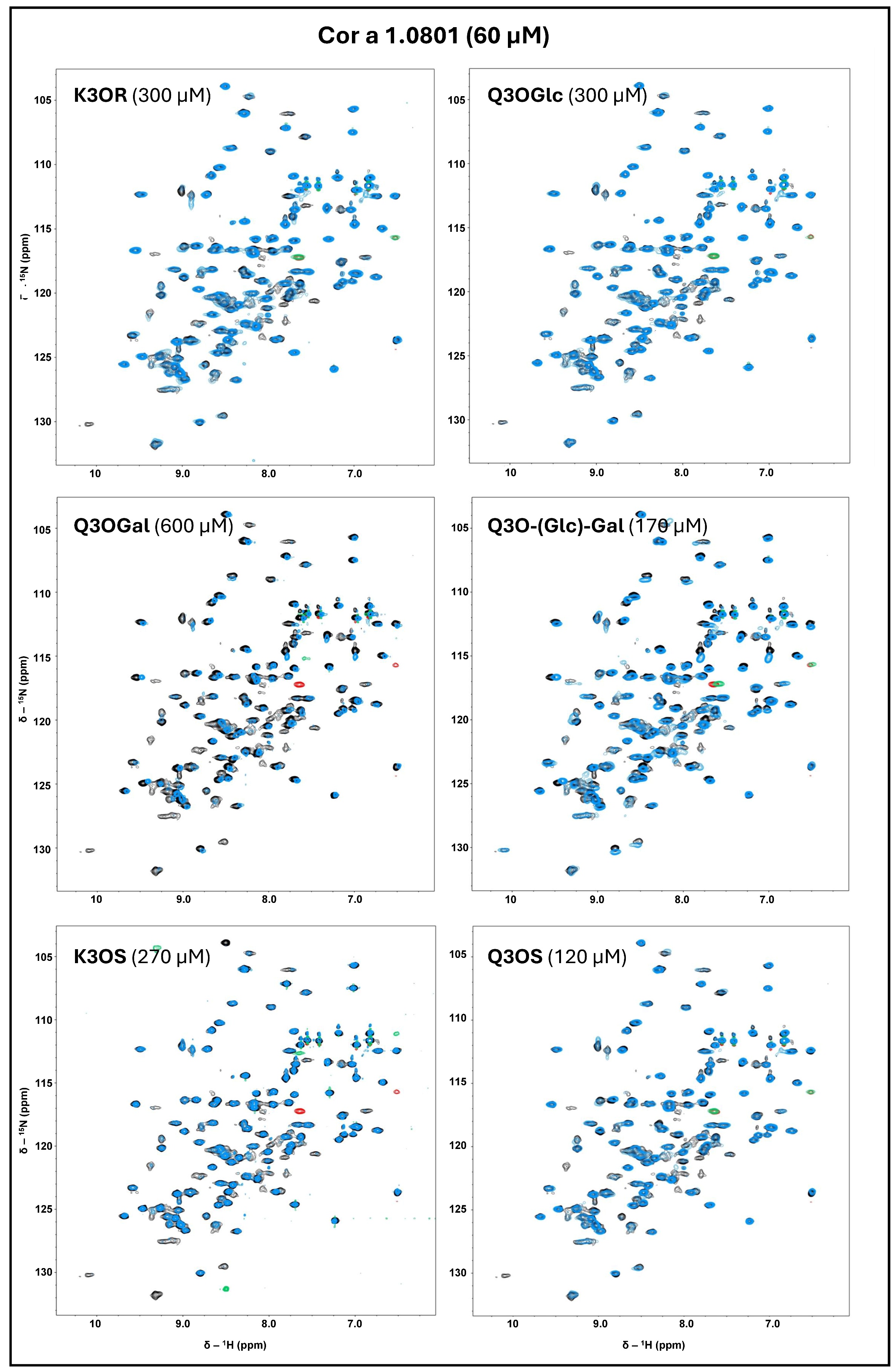


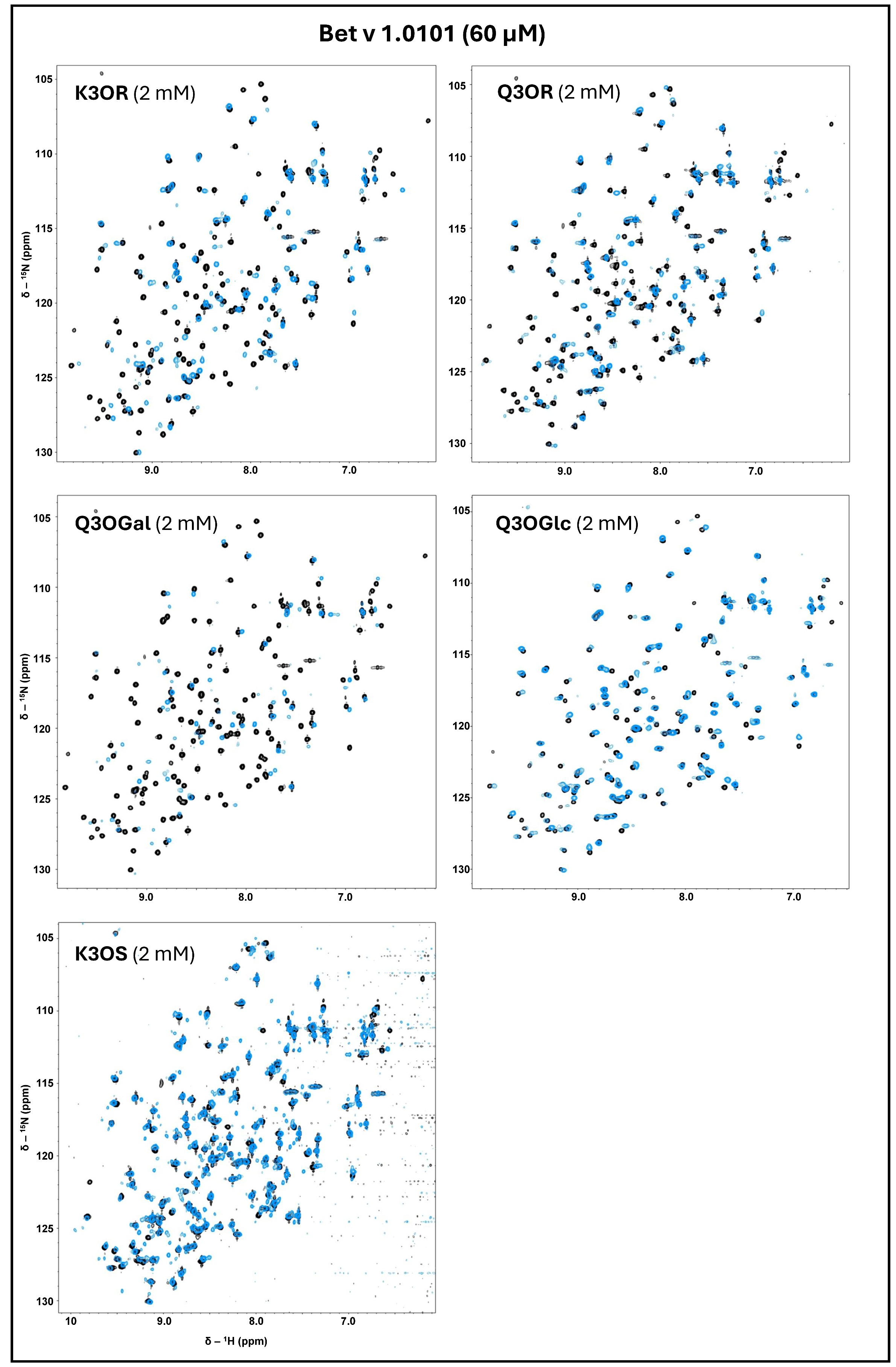


**Figure S8: Ligand screening with different Cor a 1 isoallergens using ^1^H, ^15^N HSQC spectra.** Superimposed spectra of Cor a 1 isoallergens (60 µM) without ligand (black/red) and at least a 5-fold excess of the putative ligand (blue/green). Buffer: 10 mM Na_2_HPO_4_/NaH_2_PO_4_, 50 mM NaCl, 10% ^2^H_2_O pH 7.5. Data were recorded on a Bruker Avance 900 MHz or 1GHz NMR spectrometer at 298 K.


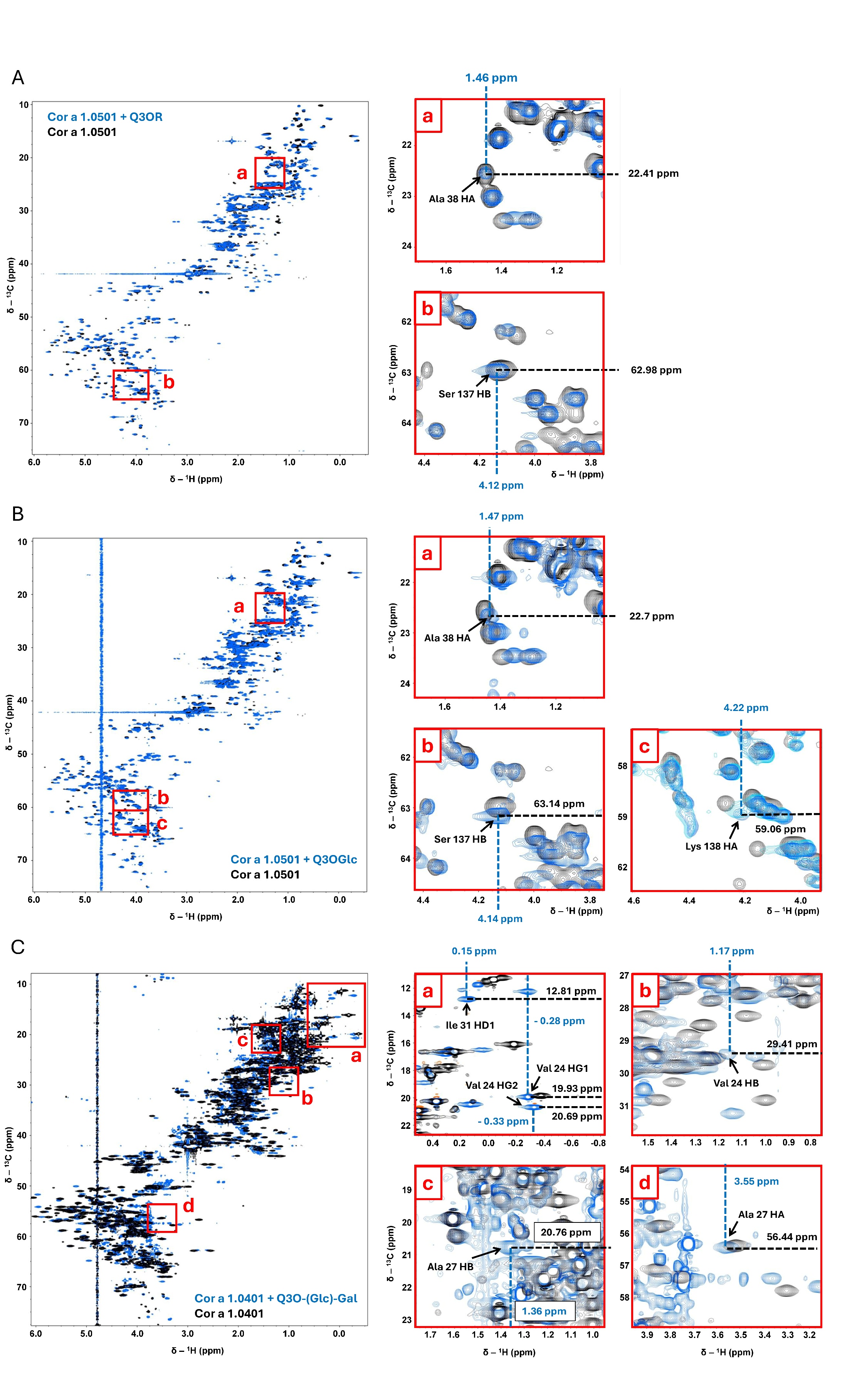


**Figure S9: Assignment of the filtered/edited NOESY signals to the side chain protons of Cor a 1.0501 in complex with Q3OR or Q3OGlc.** ^1^H, ^13^C HSQC spectrum of Cor a 1.0501 (black) in complex (blue) with **A)** Q3OR, **B)** Q3OGlc. and **C)** Q3O-(Glc)-Gal. The red boxes **a, b** and **c** show blow up sections of the spectra. The black arrows point to the signal of the assigned side chain proton. Measurements were performed at 298 or 308 K in 10 mM Na_2_HPO_4_/NaH_2_PO_4_ buffer, 50 mM NaCl at pH 7.5, 10% ^2^H_2_O, 1 mM DTT on a Bruker Avance 900 MHz or 1 GHz spectrometer.

**
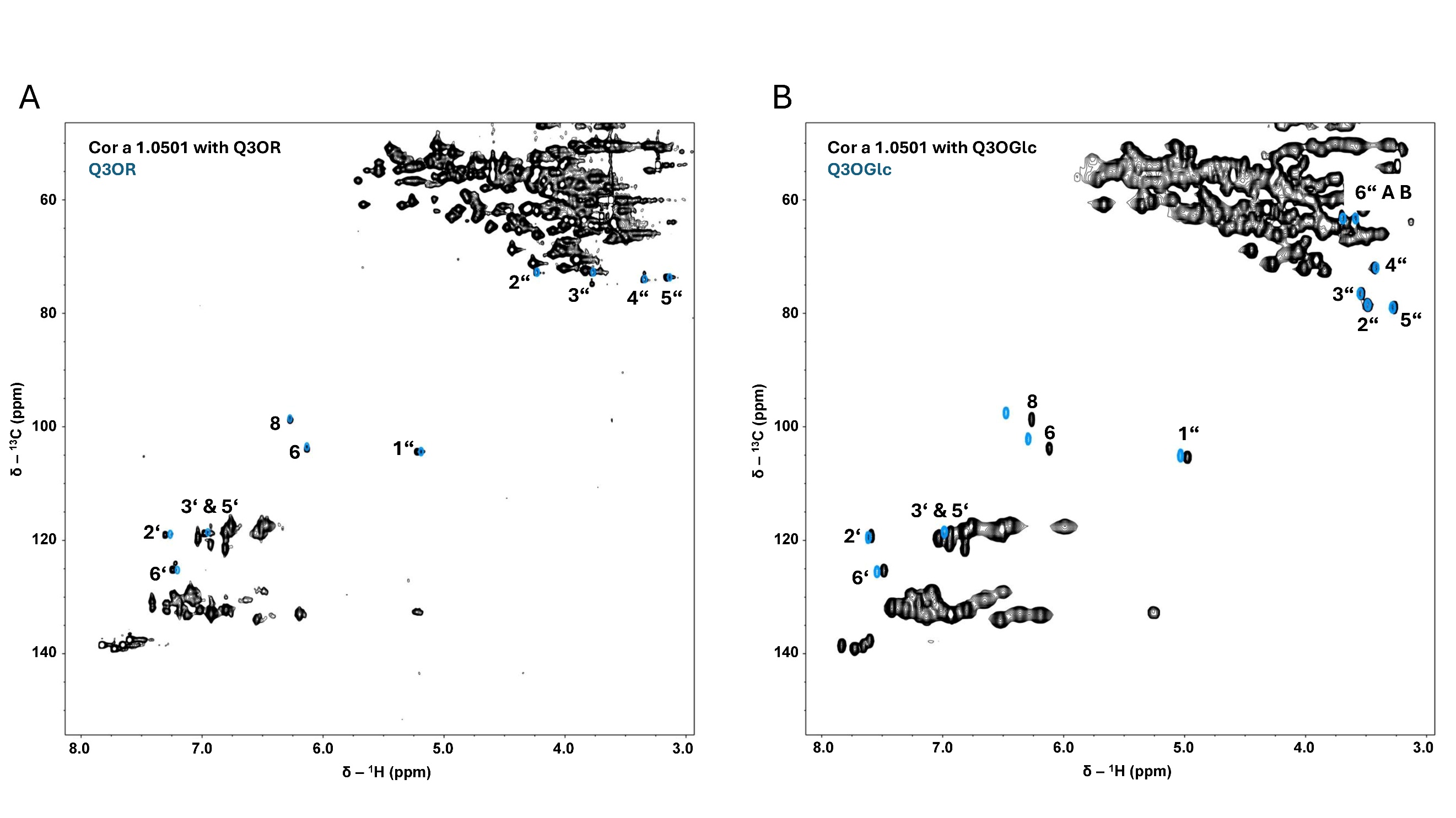
**

**Figure S10: ^1^H,^13^C-HSQC spectra of Q3OR and Q3OGlc bound to Cor a 1.0501.** Signals of the ligand in the protein/ligand complex sample (black) were identified by superimposing spectra of the ligand (blue) in the same NMR buffer (10 mM Na_2_HPO_4_/NaH_2_PO_4_ buffer, 50 mM NaCl at pH 7.5, 10% ^2^H_2_O) measured on a Bruker Avance 900 MHz or 1 GHz spectrometer at 308 K.

Cor a 1.0501 MGVHTLSDEFTSPIPAPKLFKALILDADNLLPKLLPQAIKSIETIEGDGGPGTIKKITIA 60

Cor a 1.0401_WT MGVFCYEDEATSVIPPARLFKSFVLDADNLIPKVAPQHFTSAENLEGNGGPGTIKKITFA 60

Bet v 1.0101_WT MGVFNYETETTSVIPAARLFKAFILDGDNLFPKVAPQAISSVENIEGNGGPGTIKKISFP 60

***. . * ** ** :***:::**.***:**: ** :.* *.:**:*********::

Cor a 1.0501 EGTHIKHLKHRIDAVEEEKLTYSYTLIEGDDLLDKFESISYEIKFESSPDGGAKC-TNLS 119

Cor a 1.0401_WT EGNEFKYMKHKVEEIDHANFKYCYSIIEGGPLGHTLEKISYEIKMAAAPHGGGSILKITS 120

Bet v 1.0101_WT EGFPFKYVKDRVDEVDHTNFKYNYSVIEGGPIGDTLEKISNEIKIVATPD-GGSILKISN 119

** :*::*.::: ::. ::.* *::***. : ..:*.** ***: ::*. *.. . .

Cor a 1.0501 KYHPKPGVQINEEEIKASKEKGMAVYRAVEAFLLANPEAYA 160

Cor a 1.0401_WT KYHTKGNASINEEEIKAGKEKAAGLFKAVEAYLLAHPDAYC 161

Bet v 1.0101_WT KYHTKGDHEVKAEQVKASKEMGETLLRAVESYLLAHSDAYN 160

*** * . .:: *::**.** . : :***::***: :**

**Figure S11. Sequence comparison of Cor a 1.0501, 1.0401 and Bet v 1.0101.** The amino acid S137 in Cor a 1.0501 and Bet v 1.0101_WT and G138 in Cor a 1.0401_WT are highlighted in green and turquoise, respectively.


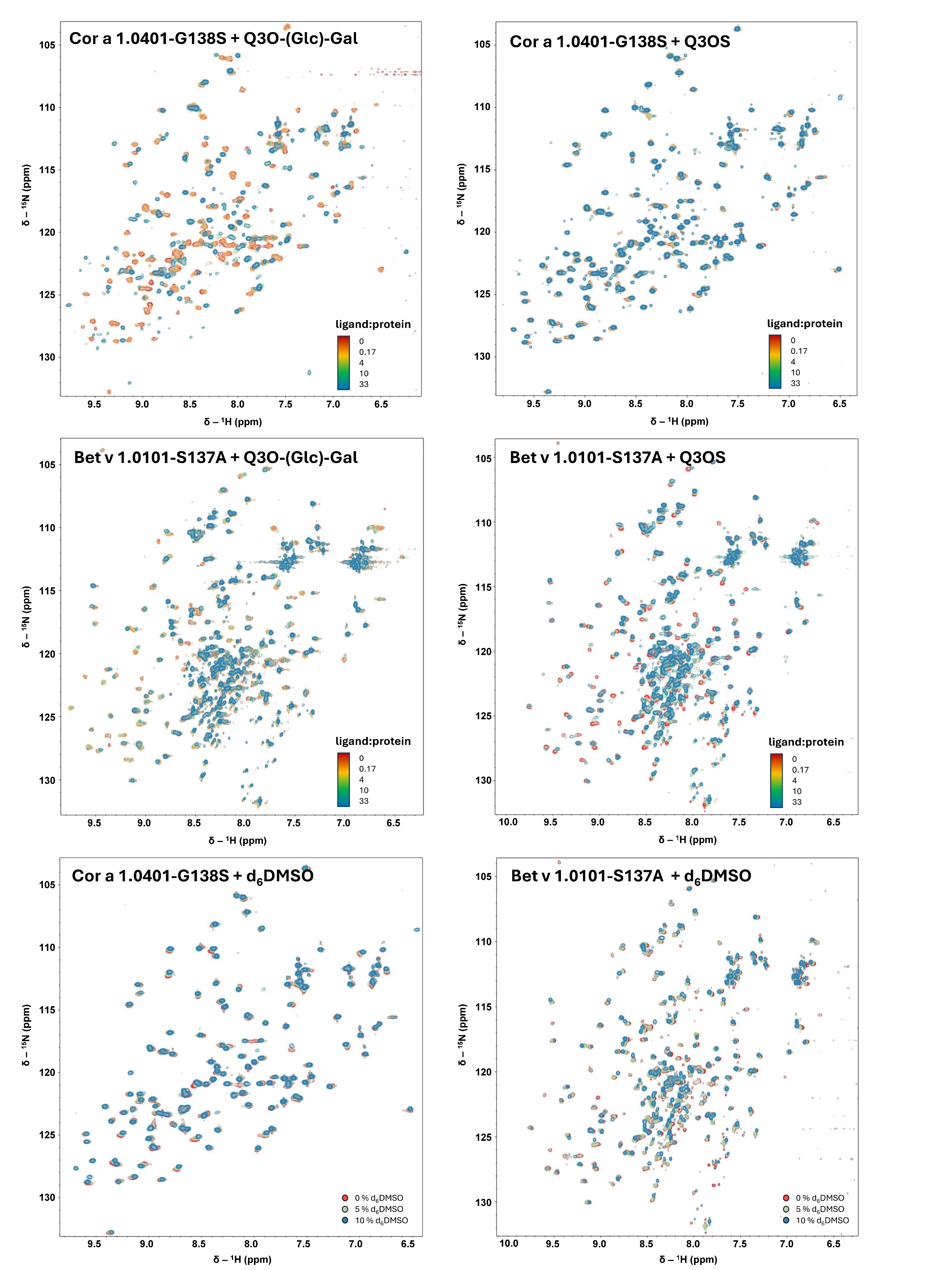


**Figure S12: ^1^H, ^15^N HSQC spectra of Cor a 1 .0401-G138S and Bet v 1.0101-S137A.** Titration of Cor a 1.0401-G138S (60 µM) and Bet v 1.0101-S137A (60 µM) with Q3O-(Glc)-Gal or Q3OS as indicated. Compounds were dissolved in 100 (v/v) d_6_DMSO. As a reference a 100 % (v/v) d6DMSO was titrated to achieve final d6DMSO concentrations of 5 and 10 % respectively. NMR buffer: 10 mM Na-phosphate pH 7.5, 1 mM EDTA 50 mM NaCl and 10 % ^2^H_2_O. ^1^H, ^15^N HSQC were recorded on a 900 MHz or 1GHz NMR spectrometer at 298K.

**Tables**

**Table S1: PDB code of Q3OR, Q3OGlc and Q3O-(Glc)-Gal**

Energy optimised PDB code of Q3OR:

HETATM 1 O1 UNL 1 1.935 -0.075 4.605 1.00 0.00 O

HETATM 2 C1 UNL 1 0.848 0.662 4.227 1.00 0.00 C

HETATM 3 C2 UNL 1 0.376 1.654 5.093 1.00 0.00 C

HETATM 4 C3 UNL 1 -0.727 2.414 4.719 1.00 0.00 C

HETATM 5 O2 UNL 1 -1.227 3.393 5.525 1.00 0.00 O

HETATM 6 C4 UNL 1 -1.360 2.199 3.501 1.00 0.00 C

HETATM 7 C5 UNL 1 0.219 0.430 2.994 1.00 0.00 C

HETATM 8 C6 UNL 1 -0.885 1.207 2.642 1.00 0.00 C

HETATM 9 O3 UNL 1 -1.567 1.061 1.462 1.00 0.00 O

HETATM 10 C7 UNL 1 0.670 -0.604 2.036 1.00 0.00 C

HETATM 11 O4 UNL 1 1.636 -1.336 2.229 1.00 0.00 O

HETATM 12 C8 UNL 1 -0.108 -0.731 0.768 1.00 0.00 C

HETATM 13 C9 UNL 1 -1.168 0.075 0.580 1.00 0.00 C

HETATM 14 C10 UNL 1 -1.999 0.021 -0.639 1.00 0.00 C

HETATM 15 C11 UNL 1 -2.186 1.188 -1.402 1.00 0.00 C

HETATM 16 C12 UNL 1 -2.966 1.167 -2.561 1.00 0.00 C

HETATM 17 C13 UNL 1 -3.560 -0.022 -2.953 1.00 0.00 C

HETATM 18 O5 UNL 1 -4.317 -0.046 -4.089 1.00 0.00 O

HETATM 19 C14 UNL 1 -2.620 -1.172 -1.047 1.00 0.00 C

HETATM 20 C15 UNL 1 -3.390 -1.184 -2.206 1.00 0.00 C

HETATM 21 O6 UNL 1 -4.001 -2.322 -2.656 1.00 0.00 O

HETATM 22 O7 UNL 1 0.244 -1.714 -0.142 1.00 0.00 O

HETATM 23 C16 UNL 1 1.564 -1.538 -0.673 1.00 0.00 C

HETATM 24 O8 UNL 1 1.500 -1.069 -2.030 1.00 0.00 O

HETATM 25 C17 UNL 1 0.885 -1.999 -2.929 1.00 0.00 C

HETATM 26 C18 UNL 1 0.845 -1.345 -4.306 1.00 0.00 C

HETATM 27 C19 UNL 1 2.341 -2.868 -0.567 1.00 0.00 C

HETATM 28 O9 UNL 1 3.739 -2.708 -0.884 1.00 0.00 O

HETATM 29 C20 UNL 1 1.760 -3.896 -1.539 1.00 0.00 C

HETATM 30 O10 UNL 1 2.599 -5.066 -1.583 1.00 0.00 O

HETATM 31 C21 UNL 1 1.652 -3.327 -2.958 1.00 0.00 C

HETATM 32 O11 UNL 1 0.991 -4.288 -3.789 1.00 0.00 O

HETATM 33 H1 UNL 1 2.235 0.182 5.491 1.00 0.00 H

HETATM 34 H2 UNL 1 0.864 1.832 6.045 1.00 0.00 H

HETATM 35 H3 UNL 1 -0.703 3.443 6.341 1.00 0.00 H

HETATM 36 H4 UNL 1 -2.221 2.801 3.223 1.00 0.00 H

HETATM 37 H5 UNL 1 -1.717 2.123 -1.102 1.00 0.00 H

HETATM 38 H6 UNL 1 -3.105 2.067 -3.151 1.00 0.00 H

HETATM 39 H7 UNL 1 -4.616 -0.974 -4.164 1.00 0.00 H

HETATM 40 H8 UNL 1 -2.494 -2.077 -0.461 1.00 0.00 H

HETATM 41 H9 UNL 1 -3.741 -3.059 -2.077 1.00 0.00 H

HETATM 42 H10 UNL 1 2.129 -0.762 -0.143 1.00 0.00 H

HETATM 43 H11 UNL 1 -0.153 -2.172 -2.622 1.00 0.00 H

HETATM 44 H12 UNL 1 1.856 -1.119 -4.662 1.00 0.00 H

HETATM 45 H13 UNL 1 0.309 -0.391 -4.257 1.00 0.00 H

HETATM 46 H14 UNL 1 0.348 -1.984 -5.042 1.00 0.00 H

HETATM 47 H15 UNL 1 2.294 -3.274 0.449 1.00 0.00 H

HETATM 48 H16 UNL 1 4.101 -2.079 -0.231 1.00 0.00 H

HETATM 49 H17 UNL 1 0.774 -4.233 -1.200 1.00 0.00 H

HETATM 50 H18 UNL 1 3.514 -4.720 -1.514 1.00 0.00 H

HETATM 51 H19 UNL 1 2.657 -3.180 -3.373 1.00 0.00 H

HETATM 52 H20 UNL 1 1.427 -5.141 -3.590 1.00 0.00 H

CONECT 1 2 33

CONECT 2 1 3 7

CONECT 3 2 4 34

CONECT 4 3 5 6

CONECT 5 4 35

CONECT 6 4 8 36

CONECT 7 2 8 10

CONECT 8 6 7 9

CONECT 9 8 13

CONECT 10 7 11 12

CONECT 11 10

CONECT 12 10 13 22

CONECT 13 9 12 14

CONECT 14 13 15 19

CONECT 15 14 16 37

CONECT 16 15 17 38

CONECT 17 16 18 20

CONECT 18 17 39

CONECT 19 14 20 40

CONECT 20 17 19 21

CONECT 21 20 41

CONECT 22 12 23

CONECT 23 22 24 27 42

CONECT 24 23 25

CONECT 25 24 26 31 43

CONECT 26 25 44 45 46

CONECT 27 23 28 29 47

CONECT 28 27 48

CONECT 29 27 30 31 49

CONECT 30 29 50

CONECT 31 25 29 32 51

CONECT 32 31 52

CONECT 33 1

CONECT 34 3

CONECT 35 5

CONECT 36 6

CONECT 37 15

CONECT 38 16

CONECT 39 18

CONECT 40 19

CONECT 41 21

CONECT 42 23

CONECT 43 25

CONECT 44 26

CONECT 45 26

CONECT 46 26

CONECT 47 27

CONECT 48 28

CONECT 49 29

CONECT 50 30

CONECT 51 31

CONECT 52 32

MASTER 0 0 0 0 0 0 0 0 52 0 52 0

END

Energy optimised PDB code of Q3OGlc:

HETATM 1 C1 UNL 1 2.064 -0.812 5.783 1.00 0.00 C

HETATM 2 C2 UNL 1 1.081 0.163 5.920 1.00 0.00 C

HETATM 3 C3 UNL 1 0.461 0.668 4.778 1.00 0.00 C

HETATM 4 C4 UNL 1 0.827 0.199 3.512 1.00 0.00 C

HETATM 5 C5 UNL 1 1.812 -0.777 3.389 1.00 0.00 C

HETATM 6 C6 UNL 1 2.434 -1.288 4.528 1.00 0.00 C

HETATM 7 O1 UNL 1 2.218 -1.280 2.179 1.00 0.00 O

HETATM 8 C7 UNL 1 1.666 -0.753 1.021 1.00 0.00 C

HETATM 9 C8 UNL 1 0.687 0.171 0.998 1.00 0.00 C

HETATM 10 C9 UNL 1 0.197 0.708 2.289 1.00 0.00 C

HETATM 11 O2 UNL 1 -0.707 1.540 2.338 1.00 0.00 O

HETATM 12 O3 UNL 1 0.150 0.709 -0.153 1.00 0.00 O

HETATM 13 C10 UNL 1 -1.046 0.023 -0.564 1.00 0.00 C

HETATM 14 O4 UNL 1 -0.739 -0.725 -1.739 1.00 0.00 O

HETATM 15 C11 UNL 1 -1.874 -1.410 -2.293 1.00 0.00 C

HETATM 16 C12 UNL 1 -2.914 -0.380 -2.743 1.00 0.00 C

HETATM 17 C13 UNL 1 -3.325 0.466 -1.538 1.00 0.00 C

HETATM 18 C14 UNL 1 -2.112 1.100 -0.857 1.00 0.00 C

HETATM 19 O5 UNL 1 -2.590 1.754 0.331 1.00 0.00 O

HETATM 20 O6 UNL 1 -4.242 1.485 -1.972 1.00 0.00 O

HETATM 21 O7 UNL 1 -4.076 -1.009 -3.287 1.00 0.00 O

HETATM 22 C15 UNL 1 -1.388 -2.271 -3.465 1.00 0.00 C

HETATM 23 O8 UNL 1 -0.617 -3.368 -2.966 1.00 0.00 O

HETATM 24 C16 UNL 1 2.232 -1.341 -0.207 1.00 0.00 C

HETATM 25 C17 UNL 1 2.205 -2.736 -0.387 1.00 0.00 C

HETATM 26 C18 UNL 1 2.719 -3.317 -1.549 1.00 0.00 C

HETATM 27 C19 UNL 1 3.267 -2.503 -2.527 1.00 0.00 C

HETATM 28 C20 UNL 1 3.341 -1.126 -2.339 1.00 0.00 C

HETATM 29 C21 UNL 1 2.815 -0.536 -1.197 1.00 0.00 C

HETATM 30 O9 UNL 1 3.964 -0.337 -3.264 1.00 0.00 O

HETATM 31 O10 UNL 1 3.702 -3.067 -3.692 1.00 0.00 O

HETATM 32 O11 UNL 1 -0.499 1.623 4.975 1.00 0.00 O

HETATM 33 O12 UNL 1 2.699 -1.336 6.870 1.00 0.00 O

HETATM 34 H1 UNL 1 0.786 0.539 6.895 1.00 0.00 H

HETATM 35 H2 UNL 1 3.204 -2.049 4.441 1.00 0.00 H

HETATM 36 H3 UNL 1 -1.406 -0.657 0.222 1.00 0.00 H

HETATM 37 H4 UNL 1 -2.289 -2.079 -1.527 1.00 0.00 H

HETATM 38 H5 UNL 1 -2.500 0.271 -3.523 1.00 0.00 H

HETATM 39 H6 UNL 1 -3.888 -0.146 -0.823 1.00 0.00 H

HETATM 40 H7 UNL 1 -1.693 1.887 -1.496 1.00 0.00 H

HETATM 41 H8 UNL 1 -1.818 1.925 0.914 1.00 0.00 H

HETATM 42 H9 UNL 1 -4.335 2.083 -1.201 1.00 0.00 H

HETATM 43 H10 UNL 1 -4.748 -0.298 -3.342 1.00 0.00 H

HETATM 44 H11 UNL 1 -0.745 -1.686 -4.132 1.00 0.00 H

HETATM 45 H12 UNL 1 -2.221 -2.689 -4.037 1.00 0.00 H

HETATM 46 H13 UNL 1 -0.134 -3.741 -3.722 1.00 0.00 H

HETATM 47 H14 UNL 1 1.759 -3.379 0.369 1.00 0.00 H

HETATM 48 H15 UNL 1 2.664 -4.392 -1.695 1.00 0.00 H

HETATM 49 H16 UNL 1 2.870 0.542 -1.080 1.00 0.00 H

HETATM 50 H17 UNL 1 4.751 -0.835 -3.553 1.00 0.00 H

HETATM 51 H18 UNL 1 3.517 -2.412 -4.389 1.00 0.00 H

HETATM 52 H19 UNL 1 -0.859 1.889 4.100 1.00 0.00 H

HETATM 53 H20 UNL 1 2.352 -0.912 7.673 1.00 0.00 H

CONECT 1 2 6 33

CONECT 2 1 3 34

CONECT 3 2 4 32

CONECT 4 3 5 10

CONECT 5 4 6 7

CONECT 6 1 5 35

CONECT 7 5 8

CONECT 8 7 9 24

CONECT 9 8 10 12

CONECT 10 4 9 11

CONECT 11 10

CONECT 12 9 13

CONECT 13 12 14 18 36

CONECT 14 13 15

CONECT 15 14 16 22 37

CONECT 16 15 17 21 38

CONECT 17 16 18 20 39

CONECT 18 13 17 19 40

CONECT 19 18 41

CONECT 20 17 42

CONECT 21 16 43

CONECT 22 15 23 44 45

CONECT 23 22 46

CONECT 24 8 25 29

CONECT 25 24 26 47

CONECT 26 25 27 48

CONECT 27 26 28 31

CONECT 28 27 29 30

CONECT 29 24 28 49

CONECT 30 28 50

CONECT 31 27 51

CONECT 32 3 52

CONECT 33 1 53

CONECT 34 2

CONECT 35 6

CONECT 36 13

CONECT 37 15

CONECT 38 16

CONECT 39 17

CONECT 40 18

CONECT 41 19

CONECT 42 20

CONECT 43 21

CONECT 44 22

CONECT 45 22

CONECT 46 23

CONECT 47 25

CONECT 48 26

CONECT 49 29

CONECT 50 30

CONECT 51 31

CONECT 52 32

CONECT 53 33

MASTER 0 0 0 0 0 0 0 0 53 0 53 0

END

Energy optimised PDB code of Q3O-(Glc)-Gal:

HETATM 1 C1 UNL 1 -2.017 2.355 1.797 1.00 0.00 C

HETATM 2 C2 UNL 1 -2.993 1.693 2.548 1.00 0.00 C

HETATM 3 C3 UNL 1 -2.643 0.558 3.261 1.00 0.00 C

HETATM 4 C4 UNL 1 -1.336 0.085 3.241 1.00 0.00 C

HETATM 5 C5 UNL 1 -0.352 0.762 2.527 1.00 0.00 C

HETATM 6 C6 UNL 1 -0.685 1.896 1.766 1.00 0.00 C

HETATM 7 C7 UNL 1 0.316 2.616 0.939 1.00 0.00 C

HETATM 8 C8 UNL 1 1.220 1.982 0.163 1.00 0.00 C

HETATM 9 C9 UNL 1 2.138 2.804 -0.668 1.00 0.00 C

HETATM 10 O1 UNL 1 3.005 2.315 -1.387 1.00 0.00 O

HETATM 11 C10 UNL 1 1.966 4.260 -0.614 1.00 0.00 C

HETATM 12 C11 UNL 1 2.757 5.124 -1.381 1.00 0.00 C

HETATM 13 C12 UNL 1 2.562 6.502 -1.317 1.00 0.00 C

HETATM 14 C13 UNL 1 1.574 7.007 -0.478 1.00 0.00 C

HETATM 15 C14 UNL 1 0.784 6.159 0.292 1.00 0.00 C

HETATM 16 C15 UNL 1 0.982 4.780 0.221 1.00 0.00 C

HETATM 17 O2 UNL 1 0.171 3.995 0.996 1.00 0.00 O

HETATM 18 O3 UNL 1 1.344 8.347 -0.380 1.00 0.00 O

HETATM 19 O4 UNL 1 3.743 4.684 -2.223 1.00 0.00 O

HETATM 20 O5 UNL 1 1.329 0.606 0.132 1.00 0.00 O

HETATM 21 C16 UNL 1 1.132 0.001 -1.168 1.00 0.00 C

HETATM 22 C17 UNL 1 0.599 -1.426 -0.863 1.00 0.00 C

HETATM 23 C18 UNL 1 0.027 -2.118 -2.115 1.00 0.00 C

HETATM 24 C19 UNL 1 -0.786 -1.167 -3.006 1.00 0.00 C

HETATM 25 C20 UNL 1 -0.001 0.122 -3.249 1.00 0.00 C

HETATM 26 O6 UNL 1 0.245 0.763 -1.989 1.00 0.00 O

HETATM 27 C21 UNL 1 -0.766 1.120 -4.120 1.00 0.00 C

HETATM 28 O7 UNL 1 0.077 2.229 -4.430 1.00 0.00 O

HETATM 29 O8 UNL 1 -2.029 -0.922 -2.318 1.00 0.00 O

HETATM 30 O9 UNL 1 -0.873 -3.176 -1.718 1.00 0.00 O

HETATM 31 O10 UNL 1 1.717 -2.137 -0.276 1.00 0.00 O

HETATM 32 C22 UNL 1 1.499 -3.423 0.310 1.00 0.00 C

HETATM 33 C23 UNL 1 0.418 -3.383 1.408 1.00 0.00 C

HETATM 34 C24 UNL 1 0.265 -4.772 2.018 1.00 0.00 C

HETATM 35 C25 UNL 1 0.045 -5.821 0.921 1.00 0.00 C

HETATM 36 C26 UNL 1 1.186 -5.746 -0.116 1.00 0.00 C

HETATM 37 O11 UNL 1 1.283 -4.422 -0.669 1.00 0.00 O

HETATM 38 C27 UNL 1 0.966 -6.715 -1.288 1.00 0.00 C

HETATM 39 O12 UNL 1 1.415 -8.026 -0.970 1.00 0.00 O

HETATM 40 O13 UNL 1 -0.046 -7.103 1.543 1.00 0.00 O

HETATM 41 O14 UNL 1 -0.854 -4.802 2.921 1.00 0.00 O

HETATM 42 O15 UNL 1 0.765 -2.427 2.439 1.00 0.00 O

HETATM 43 O16 UNL 1 -1.064 -1.069 3.925 1.00 0.00 O

HETATM 44 O17 UNL 1 -3.599 -0.102 3.977 1.00 0.00 O

HETATM 45 H1 UNL 1 -2.311 3.235 1.227 1.00 0.00 H

HETATM 46 H2 UNL 1 -4.017 2.054 2.566 1.00 0.00 H

HETATM 47 H3 UNL 1 0.674 0.413 2.564 1.00 0.00 H

HETATM 48 H4 UNL 1 3.184 7.155 -1.921 1.00 0.00 H

HETATM 49 H5 UNL 1 0.017 6.572 0.942 1.00 0.00 H

HETATM 50 H6 UNL 1 1.960 8.817 -0.968 1.00 0.00 H

HETATM 51 H7 UNL 1 3.788 3.703 -2.161 1.00 0.00 H

HETATM 52 H8 UNL 1 2.116 -0.062 -1.651 1.00 0.00 H

HETATM 53 H9 UNL 1 -0.188 -1.327 -0.108 1.00 0.00 H

HETATM 54 H10 UNL 1 0.840 -2.573 -2.691 1.00 0.00 H

HETATM 55 H11 UNL 1 -1.038 -1.669 -3.947 1.00 0.00 H

HETATM 56 H12 UNL 1 0.964 -0.093 -3.726 1.00 0.00 H

HETATM 57 H13 UNL 1 -1.079 0.664 -5.063 1.00 0.00 H

HETATM 58 H14 UNL 1 -1.646 1.518 -3.605 1.00 0.00 H

HETATM 59 H15 UNL 1 0.443 2.541 -3.581 1.00 0.00 H

HETATM 60 H16 UNL 1 -2.616 -0.417 -2.908 1.00 0.00 H

HETATM 61 H17 UNL 1 -1.735 -2.734 -1.563 1.00 0.00 H

HETATM 62 H18 UNL 1 2.459 -3.649 0.793 1.00 0.00 H

HETATM 63 H19 UNL 1 -0.553 -3.068 1.014 1.00 0.00 H

HETATM 64 H20 UNL 1 1.142 -5.030 2.625 1.00 0.00 H

HETATM 65 H21 UNL 1 -0.919 -5.630 0.434 1.00 0.00 H

HETATM 66 H22 UNL 1 2.139 -6.001 0.366 1.00 0.00 H

HETATM 67 H23 UNL 1 1.524 -6.379 -2.169 1.00 0.00 H

HETATM 68 H24 UNL 1 -0.093 -6.755 -1.569 1.00 0.00 H

HETATM 69 H25 UNL 1 1.076 -8.620 -1.661 1.00 0.00 H

HETATM 70 H26 UNL 1 -0.677 -6.989 2.282 1.00 0.00 H

HETATM 71 H27 UNL 1 -0.742 -4.039 3.519 1.00 0.00 H

HETATM 72 H28 UNL 1 1.352 -1.779 1.990 1.00 0.00 H

HETATM 73 H29 UNL 1 -0.257 -1.469 3.534 1.00 0.00 H

HETATM 74 H30 UNL 1 -3.119 -0.839 4.405 1.00 0.00 H

CONECT 1 2 6 45

CONECT 2 1 3 46

CONECT 3 2 4 44

CONECT 4 3 5 43

CONECT 5 4 6 47

CONECT 6 1 5 7

CONECT 7 6 8 17

CONECT 8 7 9 20

CONECT 9 8 10 11

CONECT 10 9

CONECT 11 9 12 16

CONECT 12 11 13 19

CONECT 13 12 14 48

CONECT 14 13 15 18

CONECT 15 14 16 49

CONECT 16 11 15 17

CONECT 17 7 16

CONECT 18 14 50

CONECT 19 12 51

CONECT 20 8 21

CONECT 21 20 22 26 52

CONECT 22 21 23 31 53

CONECT 23 22 24 30 54

CONECT 24 23 25 29 55

CONECT 25 24 26 27 56

CONECT 26 21 25

CONECT 27 25 28 57 58

CONECT 28 27 59

CONECT 29 24 60

CONECT 30 23 61

CONECT 31 22 32

CONECT 32 31 33 37 62

CONECT 33 32 34 42 63

CONECT 34 33 35 41 64

CONECT 35 34 36 40 65

CONECT 36 35 37 38 66

CONECT 37 32 36

CONECT 38 36 39 67 68

CONECT 39 38 69

CONECT 40 35 70

CONECT 41 34 71

CONECT 42 33 72

CONECT 43 4 73

CONECT 44 3 74

CONECT 45 1

CONECT 46 2

CONECT 47 5

CONECT 48 13

CONECT 49 15

CONECT 50 18

CONECT 51 19

CONECT 52 21

CONECT 53 22

CONECT 54 23

CONECT 55 24

CONECT 56 25

CONECT 57 27

CONECT 58 27

CONECT 59 28

CONECT 60 29

CONECT 61 30

CONECT 62 32

CONECT 63 33

CONECT 64 34

CONECT 65 35

CONECT 66 36

CONECT 67 38

CONECT 68 38

CONECT 69 39

CONECT 70 40

CONECT 71 41

CONECT 72 42

CONECT 73 43

CONECT 74 44

MASTER 0 0 0 0 0 0 0 0 74 0 74 0

END

**Table S2: Distance restraints used for HADDOCK**

Distance restraints for Cor a 1.0501 and Q3OR:

ASSIGN (segid A and resid 38 and name CB) (segid B and resid 1 and name C12) 7.0 7.0 0.0

ASSIGN (segid A and resid 137 and name CB) (segid B and resid 1 and name C21) 7.0 7.0 0.0

Distance restraints for Cor a 1.0501 and Q3OGlc:

ASSIGN (segid A and resid 38 and name CB) (segid B and resid 1 and name C18) 7.0 7.0 0.0
ASSIGN (segid A and resid 138 and name CA) (segid B and resid 1 and name C15) 5.0 5.0 0.0
ASSIGN (segid A and resid 137 and name CB) (segid B and resid 1 and name C12) 6.0 6.0 0.0

Distance restraints for Cor a 1.0401 and Q3O-(Glc)-Gal:

ASSIGN (segid A and resid 24 and name CG2) (segid B and resid 1 and name C12) 5.0 5.0 0.0

ASSIGN (segid A and resid 27 and name CG2) (segid B and resid 1 and name C19) 5.0 5.0 0.0

ASSIGN (segid A and resid 31 and name CG2) (segid B and resid 1 and name C24) 5.0 5.0 0.0

**Table S3: Partial amino acid side chain assignment of Cor a 1.0401-3CS in complex with Q3O-(Glc)-Gal**

| **Amino acid number** | **Amino acid** | **Proton type** | **Number of protons** | **Hetero nuclei** | **δ-1H**  **(ppm)** | **δ-13C, δ-15N**  **(ppm)** |
| --- | --- | --- | --- | --- | --- | --- |
| 3 | VAL | HA | 1 | C | 4.832 | 61.401 |
| 3 | VAL | HB | 1 | C | 1.618 | 33.583 |
| 3 | VAL | HG1* | 3 | C | 0.374 | 20.240 |
| 3 | VAL | HG2* | 3 | C | 0.491 | 20.937 |
| 4 | PHE | HN | 1 | N | 8.912 | 128.662 |
| 5 | SER | HN | 1 | N | 8.354 | 118.769 |
| 6 | TYR | HN | 1 | N | 9.274 | 123.221 |
| 7 | GLU | HN | 1 | N | 8.527 | 121.258 |
| 8 | ASP | HN | 1 | N | 9.143 | 123.395 |
| 10 | ALA | HB* | 3 | C | 1.412 | 22.899 |
| 11 | THR | HA | 1 | C | 5.054 | 59.920 |
| 11 | THR | HB | 1 | C | 4.265 | 71.755 |
| 11 | THR | HG2* | 3 | C | 1.211 | 21.716 |
| 12 | SER | HN | 1 | N | 8.370 | 112.414 |
| 13 | VAL | HN | 1 | N | 8.226 | 120.824 |
| 13 | VAL | HA | 1 | C | 4.380 | 62.795 |
| 13 | VAL | HB | 1 | C | 2.258 | 31.843 |
| 13 | VAL | HG1* | 3 | C | 0.622 | 21.316 |
| 13 | VAL | HG2* | 3 | C | 0.700 | 21.834 |
| 14 | ILE | HN | 1 | N | 8.346 | 127.504 |
| 14 | ILE | HD1* | 3 | C | 0.784 | 10.634 |
| 17 | ALA | HN | 1 | N | 8.336 | 117.169 |
| 17 | ALA | HB* | 3 | C | 1.540 | 17.934 |
| 18 | ARG | HN | 1 | N | 6.865 | 116.352 |
| 19 | LEU | HN | 1 | N | 7.865 | 121.978 |
| 20 | PHE | HN | 1 | N | 9.104 | 118.729 |
| 22 | SER | HN | 1 | N | 6.997 | 110.507 |
| 23 | PHE | HN | 1 | N | 8.247 | 121.701 |
| 24 | VAL | HA | 1 | C | 3.700 | 63.382 |
| 24 | VAL | HB | 1 | C | 1.167 | 29.408 |
| 24 | VAL | HG1* | 3 | C | -0.282 | 19.935 |
| 24 | VAL | HG2* | 3 | C | -0.326 | 20.692 |
| 25 | LEU | HN | 1 | N | 7.571 | 114.772 |
| 26 | ASP | HN | 1 | N | 7.084 | 118.519 |
| 27 | ALA | HN | 1 | N | 6.258 | 121.611 |
| 27 | ALA | HA | 1 | C | 3.554 | 56.439 |
| 27 | ALA | HB* | 3 | C | 1.359 | 20.764 |
| 28 | ASP | HN | 1 | N | 8.123 | 113.696 |
| 29 | ASN | HN | 1 | N | 7.155 | 112.134 |
| 30 | LEU | HN | 1 | N | 8.329 | 120.270 |
| 30 | LEU | HA | 1 | C | 3.966 | 57.668 |
| 30 | LEU | HB1 | 1 | C | 1.336 | 43.457 |
| 30 | LEU | HB2 | 1 | C | 1.502 | 43.428 |
| 30 | LEU | HG | 1 | C | 1.072 | 26.685 |
| 30 | LEU | HD1* | 3 | C | 0.449 | 26.769 |
| 30 | LEU | HD2* | 3 | C | 0.516 | 23.026 |
| 31 | ILE | HD1* | 3 | C | 0.146 | 12.812 |
| 33 | LYS | HN | 1 | N | 6.694 | 113.170 |
| 34 | VAL | HG1* | 3 | C | 0.953 | 19.106 |
| 34 | VAL | HG2* | 3 | C | 1.069 | 21.356 |
| 42 | ALA | HN | 1 | N | 8.697 | 124.708 |
| 42 | ALA | HA | 1 | C | 5.078 | 51.605 |
| 42 | ALA | HB* | 3 | C | 1.240 | 22.871 |
| 43 | GLU | HN | 1 | N | 8.453 | 118.752 |
| 44 | ASN | HN | 1 | N | 9.124 | 122.922 |
| 45 | LEU | HN | 1 | N | 9.254 | 127.495 |
| 45 | LEU | HB1 | 1 | C | 1.626 | 42.351 |
| 45 | LEU | HB2 | 1 | C | 1.401 | 42.083 |
| 45 | LEU | HG | 1 | C | 1.580 | 26.848 |
| 45 | LEU | HD1* | 3 | C | 0.812 | 25.457 |
| 45 | LEU | HD2* | 3 | C | 0.756 | 22.547 |
| 46 | GLU | HN | 1 | N | 7.676 | 116.450 |
| 47 | GLY | HN | 1 | N | 8.471 | 110.413 |
| 48 | ASN | HN | 1 | N | 8.220 | 114.149 |
| 49 | GLY | HN | 1 | N | 8.800 | 110.258 |
| 50 | GLY | HN | 1 | N | 8.008 | 126.507 |
| 52 | GLY | HN | 1 | N | 9.115 | 114.511 |
| 53 | THR | HN | 1 | N | 7.634 | 118.075 |
| 53 | THR | HA | 1 | C | 4.554 | 64.484 |
| 53 | THR | HB | 1 | C | 3.949 | 69.886 |
| 54 | ILE | HN | 1 | N | 8.366 | 125.703 |
| 54 | ILE | HA | 1 | C | 4.606 | 60.592 |
| 54 | ILE | HG2* | 3 | C | 0.813 | 17.516 |
| 54 | ILE | HD1* | 3 | C | 0.858 | 12.682 |
| 55 | LYS | HN | 1 | N | 9.483 | 128.540 |
| 56 | LYS | HN | 1 | N | 9.334 | 124.046 |
| 57 | ILE | HN | 1 | N | 9.093 | 111.182 |
| 57 | ILE | HA | 1 | C | 4.721 | 59.611 |
| 57 | ILE | HB | 1 | C | 1.218 | 39.618 |
| 57 | ILE | HG11 | 1 | C | 0.432 | 18.126 |
| 57 | ILE | HG12 | 1 | C | 0.432 | 18.126 |
| 57 | ILE | HG2* | 3 | C | 0.432 | 18.126 |
| 57 | ILE | HD1* | 3 | C | -0.277 | 12.233 |
| 70 | HIS | HN | 1 | N | 9.343 | 122.830 |
| 72 | VAL | HN | 1 | N | 9.221 | 128.774 |
| 72 | VAL | HA | 1 | C | 3.871 | 64.299 |
| 72 | VAL | HB | 1 | C | 2.170 | 32.118 |
| 72 | VAL | HG1* | 3 | C | 1.026 | 20.706 |
| 72 | VAL | HG2* | 3 | C | 0.929 | 20.500 |
| 75 | ILE | HN | 1 | N | 8.756 | 123.995 |
| 75 | ILE | HA | 1 | C | 4.331 | 62.088 |
| 75 | ILE | HG2* | 3 | C | 0.853 | 17.479 |
| 75 | ILE | HD1* | 3 | C | 0.879 | 13.422 |
| 76 | ASP | HN | 1 | N | 9.445 | 128.040 |
| 78 | ALA | HN | 1 | N | 8.048 | 119.370 |
| 78 | ALA | HA | 1 | C | 3.787 | 54.156 |
| 78 | ALA | HB* | 3 | C | 1.302 | 18.501 |
| 79 | ASN | HN | 1 | N | 7.069 | 111.412 |
| 80 | PHE | HN | 1 | N | 7.212 | 117.362 |
| 81 | LYS | HN | 1 | N | 7.995 | 117.030 |
| 84 | TYR | HN | 1 | N | 8.750 | 121.498 |
| 85 | SER | HN | 1 | N | 9.409 | 113.060 |
| 86 | ILE | HN | 1 | N | 9.812 | 125.902 |
| 88 | GLU | HN | 1 | N | 7.666 | 122.327 |
| 89 | GLY | HN | 1 | N | 9.002 | 113.167 |
| 92 | LEU | HN | 1 | N | 7.342 | 119.349 |
| 92 | LEU | HB1 | 1 | C | 1.581 | 39.751 |
| 92 | LEU | HB2 | 1 | C | 1.602 | 39.757 |
| 92 | LEU | HG | 1 | C | 1.702 | 26.858 |
| 92 | LEU | HD1* | 3 | C | 0.678 | 25.245 |
| 92 | LEU | HD2* | 3 | C | 0.564 | 22.822 |
| 93 | GLY | HN | 1 | N | 7.459 | 124.909 |
| 95 | THR | HA | 1 | C | 4.454 | 62.811 |
| 95 | THR | HB | 1 | C | 4.319 | 70.184 |
| 95 | THR | HG2* | 3 | C | 1.096 | 22.067 |
| 97 | GLU | HN | 1 | N | 9.216 | 121.024 |
| 98 | LYS | HN | 1 | N | 8.050 | 118.173 |
| 99 | ILE | HN | 1 | N | 7.682 | 122.669 |
| 99 | ILE | HA | 1 | C | 4.782 | 60.763 |
| 99 | ILE | HB | 1 | C | 1.223 | 42.656 |
| 99 | ILE | HG11 | 1 | C | 1.454 | 27.634 |
| 99 | ILE | HG12 | 1 | C | 1.070 | 27.644 |
| 99 | ILE | HG2* | 3 | C | 0.034 | 16.508 |
| 99 | ILE | HD1* | 3 | C | 0.825 | 14.773 |
| 102 | GLU | HN | 1 | N | 8.532 | 121.142 |
| 103 | ILE | HA | 1 | C | 5.066 | 60.763 |
| 103 | ILE | HB | 1 | C | 0.881 | 40.304 |
| 103 | ILE | HG11 | 1 | C | 1.310 | 29.530 |
| 103 | ILE | HG12 | 1 | C | 1.042 | 29.481 |
| 103 | ILE | HG2* | 3 | C | 0.779 | 18.781 |
| 103 | ILE | HD1* | 3 | C | 0.776 | 14.747 |
| 104 | LYS | HN | 1 | N | 8.548 | 125.674 |
| 105 | MET | HN | 1 | N | 9.050 | 123.638 |
| 106 | ALA | HA | 1 | C | 4.714 | 50.460 |
| 106 | ALA | HB* | 3 | C | 1.414 | 22.525 |
| 107 | ALA | HN | 1 | N | 8.792 | 123.298 |
| 107 | ALA | HA | 1 | C | 4.319 | 52.373 |
| 107 | ALA | HB* | 3 | C | 1.415 | 18.384 |
| 108 | ALA | HN | 1 | N | 8.010 | 126.329 |
| 108 | ALA | HA | 1 | C | 4.313 | 50.262 |
| 108 | ALA | HB* | 3 | C | 1.183 | 18.153 |
| 112 | GLY | HN | 1 | N | 8.336 | 128.774 |
| 113 | GLY | HN | 1 | N | 8.079 | 127.807 |
| 114 | SER | HN | 1 | N | 9.025 | 118.837 |
| 115 | ILE | HN | 1 | N | 9.234 | 122.899 |
| 115 | ILE | HD1* | 3 | C | 0.852 | 14.091 |
| 116 | LEU | HN | 1 | N | 9.280 | 128.006 |
| 118 | ILE | HN | 1 | N | 9.094 | 127.886 |
| 119 | THR | HA | 1 | C | 5.337 | 61.945 |
| 119 | THR | HB | 1 | C | 4.176 | 69.979 |
| 119 | THR | HG2* | 3 | C | 1.173 | 21.595 |
| 122 | TYR | HN | 1 | N | 9.565 | 124.628 |
| 123 | HIS | HN | 1 | N | 8.704 | 123.505 |
| 124 | THR | HN | 1 | N | 9.398 | 116.018 |
| 124 | THR | HA | 1 | C | 5.005 | 60.395 |
| 124 | THR | HB | 1 | C | 4.424 | 70.830 |
| 124 | THR | HG2* | 3 | C | 1.200 | 22.944 |
| 128 | ALA | HN | 1 | N | 7.818 | 122.563 |
| 128 | ALA | HA | 1 | C | 4.368 | 52.989 |
| 128 | ALA | HB* | 3 | C | 1.607 | 19.876 |
| 130 | ILE | HD1* | 3 | C | 0.588 | 13.561 |
| 131 | ASN | HN | 1 | N | 8.614 | 124.278 |
| 133 | GLU | HN | 1 | N | 8.403 | 118.707 |
| 135 | ILE | HD1* | 3 | C | 0.073 | 11.740 |
| 137 | ALA | HA | 1 | C | 4.202 | 55.017 |
| 137 | ALA | HB* | 3 | C | 1.500 | 17.954 |
| 138 | GLY | HN | 1 | N | 8.113 | 127.487 |
| 143 | ALA | HA | 1 | C | 4.202 | 55.017 |
| 143 | ALA | HB* | 3 | C | 1.518 | 18.140 |
| 147 | LYS | HN | 1 | N | 7.921 | 116.696 |
| 149 | VAL | HN | 1 | N | 8.928 | 117.271 |
| 149 | VAL | HA | 1 | C | 3.430 | 66.820 |
| 149 | VAL | HB | 1 | C | 1.541 | 30.839 |
| 149 | VAL | HG1* | 3 | C | 0.462 | 23.068 |
| 149 | VAL | HG2* | 3 | C | 0.130 | 20.487 |
| 152 | TYR | HN | 1 | N | 7.890 | 120.550 |
| 153 | LEU | HN | 1 | N | 8.567 | 120.744 |
| 153 | LEU | HA | 1 | C | 4.016 | 57.577 |
| 153 | LEU | HD1* | 3 | C | 0.951 | 22.318 |
| 153 | LEU | HD2* | 3 | C | 0.729 | 27.442 |
| 154 | LEU | HN | 1 | N | 8.353 | 120.100 |
| 155 | ALA | HA | 1 | C | 4.104 | 52.747 |
| 155 | ALA | HB* | 3 | C | 1.219 | 18.570 |
| 156 | HIS | HN | 1 | N | 7.189 | 115.568 |
| 158 | ASP | HN | 1 | N | 8.625 | 114.253 |
| 159 | ALA | HN | 1 | N | 7.848 | 125.111 |
| 161 | SER | HN | 1 | N | 7.518 | 118.666 |
